# Ethnobotany and Medicinal Potential of Wild Edible Fruit Species in Kut Chum District, Yasothon Province, Thailand

**DOI:** 10.3390/biology15090711

**Published:** 2026-04-30

**Authors:** Tammanoon Jitpromma, Piyaporn Saensouk, Santi Watthana, Surapon Saensouk

**Affiliations:** 1 Diversity of Family Zingiberaceae and Vascular Plant for Its Applications Research Unit, Mahasarakham University, Maha Sarakham 44150, Thailand; jitpromma.t@gmail.com (T.J.); pcornukaempferia@yahoo.com (P.S.); 2 Walai Rukhavej Botanical Research Institute, Mahasarakham University, Maha Sarakham 44150, Thailand; 3 Department of Biology, Faculty of Science, Mahasarakham University, Maha Sarakham 44150, Thailand; 4 School of Biology, Institute of Science, Suranaree University of Technology, Nakhon Ratchasima 30000, Thailand; santiqsbg@gmail.com

**Keywords:** ethnomedicine, medicinal plants, traditional knowledge, traditional utilization, wild edible plants, Yasothon Province

## Abstract

Wild edible fruits play an important role in supporting food security, nutrition, and traditional knowledge systems in rural communities, yet their diversity and uses remain insufficiently documented in many parts of Thailand. This study aimed to investigate the diversity, utilization, and ethnobotanical significance of wild edible fruit species in Kut Chum District, Yasothon Province. Ethnobotanical data were collected through semi-structured interviews and field surveys with local informants, and quantitative indices, including the Cultural Importance Index (CI), Fidelity Level (%FL), and Informant Consensus Factor (ICF), were applied to evaluate species significance and medicinal agreement. A total of 71 species belonging to 33 families were recorded, with most species consumed as fresh fruits and a subset used for medicinal purposes. Several species, such as *Irvingia malayana* Oliv. ex A.W.Benn., *Phyllanthus emblica* L., and *Syzygium cumini* (L.) Skeels exhibited high cultural importance, reflecting their key roles in local diets. High ICF values across therapeutic categories indicated strong consensus in ethnomedicinal knowledge. Additionally, 44 species not used medicinally in the study area were reported as medicinal in other regions, highlighting spatial variation in knowledge systems. These findings emphasize the importance of wild edible fruits as multifunctional resources contributing to food and nutritional security. Integrating culturally important species into conservation and sustainable use strategies may support biodiversity preservation and the continuity of traditional ecological knowledge.

## Simple Summary

Wild edible fruits are an important but often overlooked source of food and health benefits for rural communities. However, knowledge about these plants is gradually declining due to social and environmental changes. This study aimed to document the diversity of wild edible fruits and understand how local people in Kut Chum District, Yasothon Province, use them in their daily lives. Information was collected through interviews with community members and field observations. The study recorded 71 species of wild edible fruits, most of which are eaten fresh, and some are also used in traditional remedies. Certain species were found to be especially important because they are widely used and valued for both nutrition and health. The results also showed that some plants known as medicinal in other areas are not used that way locally, highlighting differences in knowledge between communities. Overall, this study demonstrates that wild edible fruits play a key role in supporting food availability, nutrition, and cultural traditions. Protecting these plants and the knowledge associated with them can help strengthen food security and preserve valuable cultural heritage for future generations.

## 1. Introduction

Traditional ethnobotanical knowledge represents a dynamic biocultural system shaped through long-term interactions between human societies and local plant resources [1]. This knowledge encompasses cumulative experiences related to food selection, medicinal use, harvesting practices, and preparation methods that enable communities to maintain health and well-being within specific ecological and cultural contexts [2]. In many traditional societies, plant-based foods are not perceived solely as sources of nutrition but also as functional resources with preventive and therapeutic roles, forming a continuum between food and medicine [3].

Within this framework, wild edible fruits occupy a distinctive position. They are commonly consumed as seasonal foods while simultaneously being valued for their medicinal properties, such as supporting digestion, enhancing vitality, and alleviating common ailments [4]. The traditional use of wild edible fruits often reflects detailed local knowledge regarding phenology, appropriate stages of consumption, preparation techniques, and perceived health benefits [5]. Such knowledge systems provide important insights into the biological relevance of plant species and offer valuable starting points for the identification of bioactive compounds with potential pharmaceutical applications [6].

Globally, ethnobotanical research has demonstrated that many plant species traditionally used as foods or folk remedies contain diverse bioactive compounds, including phenolic compounds, flavonoids, terpenoids, organic acids, and alkaloids, which exhibit antimicrobial, anti-inflammatory, antidiabetic, and other biological activities [7,8]. Traditional knowledge has therefore played a complementary role in guiding modern drug discovery, with numerous contemporary medicines originating from plants first identified through ethnomedical practices [9]. Documenting and analyzing traditional knowledge related to wild edible fruits is thus essential not only for cultural preservation but also for advancing biological and pharmacological research [10].

Despite their importance, traditional knowledge associated with wild edible fruits is increasingly threatened by rapid socio-economic change. Urbanization, market integration, and the growing availability of commercially cultivated fruits have altered dietary preferences and consumption patterns, particularly in rural areas [11]. As standardized and year-round products replace seasonal wild resources, knowledge related to the identification, harvesting, and medicinal use of wild edible fruits is gradually eroding [12]. This loss is further accelerated by changes in land use, agricultural intensification, and reduced intergenerational transmission of ethnobotanical knowledge [13].

Thailand possesses a long-standing tradition of plant use for both food and medicine, shaped by diverse cultural influences across regions [14]. While ethnobotanical studies in Northeastern Thailand have documented a wide range of medicinal plants and edible species, research has predominantly focused on medicinal herbs or food plants [15]. In contrast, wild edible fruit species and their roles within traditional healthcare systems remain comparatively underexplored, particularly at the local and district levels. This knowledge gap limits our understanding of the full spectrum of plant-based resources contributing to community health and constrains the identification of fruit species with promising medicinal potential [16].

Yasothon Province, located in Northeastern Thailand, is characterized by agricultural landscapes interspersed with semi-natural habitats that support diverse wild plant species. In Kut Chum District, local communities continue to rely on traditional knowledge to collect and use wild edible fruits for household consumption and folk medicine. However, systematic ethnobotanical documentation of these species, their modes of use, and their perceived health benefits remains limited.

Therefore, the present study aims to document traditional knowledge related to wild edible fruit species used by local communities in Kut Chum District, Yasothon Province, Thailand. Specifically, this study seeks to (i) record the diversity of wild edible fruit species and their ethnobotanical uses, (ii) describe traditional consumption practices, preparation methods, and medicinal applications, and (iii) assess their potential medicinal relevance based on local knowledge and supporting scientific evidence. By focusing on traditional knowledge as a foundation for biological and pharmacological exploration, this study contributes to the preservation of ethnobotanical heritage and highlights wild edible fruits as promising resources for future drug discovery and health-related research.

## 2. Materials and Methods

### 2.1. Study Area Description

**Figure 1:**
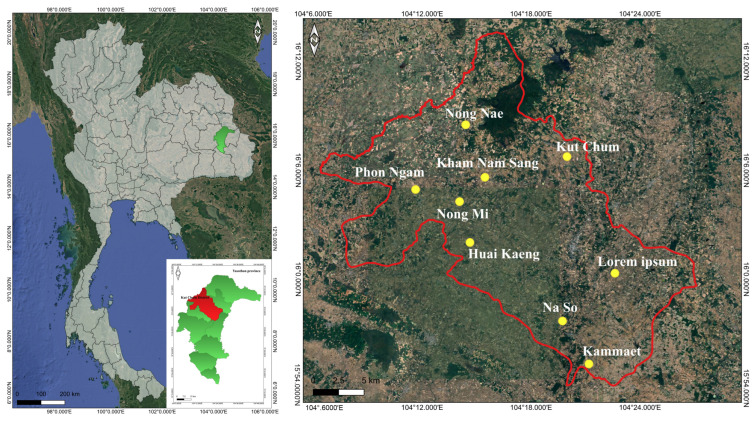
Study area map. The map on the left shows Kut Chum District highlighted in red within Yasothon Province, located in northeastern Thailand. The map on the right indicates the surveyed locations, with yellow points representing the villages visited in each sub-district during the field investigation. (map created with “QGIS” program ver. 3.34 [18], geographic system ID: WGS 84, EPSG 4326).

The study was conducted in Kut Chum District, located in Yasothon Province in Northeastern Thailand (Figure 1). The district covers approximately 544 km^2^ and lies within the Khorat Plateau, a major physiographic region characterized by predominantly flat to gently undulating terrain. Kut Chum is situated near the Chi River basin, which influences local hydrology and supports seasonal wetlands, ponds, and small streams that are important for agriculture and wild plant habitats [17].

The district experiences a tropical savanna climate (Köppen Aw) with marked seasonal variation. Annual rainfall averages about 1200–1500 mm, with the majority occurring during the monsoon season from May to October, followed by a prolonged dry season lasting approximately five to six months. Temperatures typically range from 25 to 30 °C during the cooler season (November–February) and can exceed 38 °C during the pre-monsoon period (March–May). These climatic conditions strongly affect vegetation dynamics, plant phenology, and the seasonal availability of wild edible fruits [19].

Soil in Kut Chum District is dominated by sandy loam types, particularly the Yasothon and Roi Et soil series, which are characterized by low organic matter content, limited water-holding capacity, and moderate to low fertility. These edaphic constraints restrict intensive agriculture and contribute to periodic drought stress, while low-lying areas may experience seasonal flooding [20,21]. In addition to abiotic factors, plants in these environments are exposed to various biotic pressures, including herbivorous insects such as *Nilaparvata lugens* (Stål) (Hemiptera: Delphacidae), which are widely reported as major pests in rice-based agroecosystems across Southeast Asia [22]. Plants are also affected by microbial pathogens, including fungal species such as *Magnaporthe oryzae* B.C. Couch and bacterial pathogens such as *Xanthomonas oryzae* pv. *oryzae* (*Xoo*), which are known to influence plant defense responses and ecological interactions in these systems. These biotic stresses can stimulate plant defense mechanisms and promote the production of secondary metabolites [23]. Consequently, the landscape comprises a mosaic of rain-fed rice fields, fallow lands, forest remnants, secondary vegetation, and semi-natural habitats along field margins and waterways, which together support diverse wild fruit-bearing plant species [24].

Kut Chum District has a predominantly rural population of approximately 66,000 inhabitants, most of whom engage in small-scale agriculture, especially rice cultivation. Local livelihoods remain closely connected to surrounding environments, and traditional knowledge related to wild plant use is embedded in daily subsistence practices. Wild edible fruits are commonly collected from semi-natural habitats and uncultivated areas for household consumption and traditional healthcare, making the district an appropriate setting for ethnobotanical research focused on traditional knowledge and medicinal potential.

### 2.2. Plant Collection and Identification

All wild edible fruit species reported by informants were collected through guided field walks in natural and semi-natural habitats, including forest remnants, fallow lands, field margins, and wetlands. During field collection, fertile specimens bearing fruits were preferentially sampled to ensure accurate identification. Each species was photographed in situ, and voucher specimens were prepared following standard herbarium techniques.

Collected specimens were processed, labeled, and deposited as voucher specimens at the Vascular Plant Herbarium, Mahasarakham University (VMSU), Kantharawichai District, Maha Sarakham Province, Thailand, for long-term preservation and future reference. Herbarium accession numbers were assigned to all specimens and are provided in the Supplementary Materials.

Taxonomic identification was conducted using regional and national floristic references, including the Flora of Thailand, along with relevant taxonomic literature. Scientific names, authorship, and family classifications were subsequently verified and updated according to the Plants of the World Online (POWO) [25] database to ensure nomenclatural accuracy and consistency with current taxonomic standards.

### 2.3. Ethnobotanical Data Collection

Ethnobotanical fieldwork was conducted from March 2025 to February 2026 in Kut Chum District, Yasothon Province, Northeastern Thailand. Data were collected across multiple sub-districts representing predominantly rural communities with long-standing reliance on wild plant resources. A qualitative ethnobotanical approach was employed, integrating semi-structured interviews, participant observation, and guided field walks to document traditional knowledge related to wild edible fruit species.

A total of 60 informants (30 men and 30 women), ranging in age from 25 to 65 years (Table 1), were selected using a combination of purposive sampling techniques [26]. Informants were chosen based on their recognized experience with wild plant use, including elderly villagers, traditional knowledge holders, and individuals actively involved in plant collection for food or household healthcare. This selection strategy ensured comprehensive coverage of locally relevant knowledge while capturing intergenerational perspectives. Although the sample size does not aim to be statistically representative of the total population, it is appropriate for ethnobotanical research, where emphasis is placed on depth and reliability of knowledge obtained from key informants rather than population-level generalization.

Interviews were conducted in Thai or the local dialect, depending on informant preference, and focused on ethnobotanical information related to wild edible fruit species. Data recorded included local names, parts used, modes of consumption, preparation methods, and perceived health-related or medicinal uses. Field walks with key informants allowed for direct observation of plant habitats and facilitated accurate species recognition and contextual understanding of traditional practices.

Prior to data collection, the objectives, methods, and intended outcomes of the study were clearly explained to all participants, and informed consent was obtained. The research adhered to ethical principles outlined by the International Society of Ethnobiology Code of Ethics [27] and complied with the principles of the Nagoya Protocol on Access and Benefit-Sharing [28]. Participants were informed of their rights, including voluntary participation and the freedom to withdraw from the study at any time without negative consequences.

As the study did not involve the collection of personal or sensitive data, formal institutional ethical approval was not required. Nevertheless, all research activities were conducted in accordance with internationally accepted ethical standards for ethnobotanical research, emphasizing respect, transparency, and reciprocity toward participating communities.

### 2.4. Data Analysis

#### 2.4.1. Cultural Importance Index (CI)

The Cultural Importance Index (CI) was used to assess the cultural significance of wild edible fruit species utilized by local communities in Kut Chum District, Yasothon Province, Thailand. This quantitative ethnobotanical index integrates both the frequency of use and the diversity of utilization types, allowing for a comprehensive evaluation of the relative cultural relevance of each species within the local knowledge system.

Ethnobotanical data were obtained through semi-structured interviews with local informants. All reported uses of each wild edible fruit species were recorded and classified into predefined food- and health-related utilization categories relevant to the study context. Plant species were categorized according to their primary and secondary roles within the community’s food system and traditional health practices. The classification followed standard ethnobotanical frameworks with minor modifications to reflect local dietary habits and plant-use traditions in northeastern Thailand. Each species could be assigned to one or more of the following categories [15,29]:

- Fruit: Species whose fruits are consumed fresh or fruits eaten directly, incorporated into traditional dishes, or preserved for later consumption.
- Beverage: Plant species used in the preparation of drinks, such as herbal beverages, refreshing fruit drinks, or decoctions traditionally consumed for nourishment or health benefits.
- Food ingredient or culinary additive: Species used as minor ingredients in cooking to enhance flavor, aroma, texture, or acidity in traditional dishes, such as sour fruits or plant materials added to soups, curries, or chili pastes.
- Medicinal use: Plant species utilized in traditional remedies or health-related practices, including treatments for common ailments, preventive healthcare, or functional foods believed to promote well-being according to local knowledge.

The CI was calculated following the method proposed by Tardío and Pardo-De-Santayana (2008) [30], using the following formula:

(1) $$\mathrm{CI}\;=\;\sum_{u\;=\;1}^{\mathrm{NC}}\sum_{i\;=\;1}^{N}\frac{UR_{\mathrm{ui}}}{N}$$

where CI represents the Cultural Importance Index of a given species, UR_ui_ denotes the number of use-reports for species i in use category u, NC is the total number of use categories, and N is the total number of informants interviewed.

Higher CI values indicate plant species that are frequently used across multiple use categories, reflecting multifunctionality and strong cultural embeddedness. This index highlights wild edible fruit species that play particularly important roles in daily subsistence, traditional food practices, and household healthcare, thereby identifying culturally salient species with potential relevance for further nutritional, biological, and pharmacological investigation.

#### 2.4.2. Fidelity Level (%FL)

The Fidelity Level (FL) was calculated to determine the relative healing potential of each plant species for a specific ailment, reflecting the degree of consensus among informants regarding its use. FL was computed using the following formula [31]:

(2) $$\mathrm{FL}\;=\;\frac{I_{p}}{I_{u}}\times 100$$

where I_p_ is the number of informants who independently cited the use of a species for the same specific ailment, and I_u_ is the total number of informants who mentioned the plant for any use. A higher FL value indicates a greater consensus on the use of a species for a particular therapeutic purpose.

#### 2.4.3. Informant Consensus Factor (ICF)

The Informant Consensus Factor (ICF) was used to evaluate the level of agreement among informants regarding the use of medicinal plants for specific ailment categories. It helps to identify culturally important plant uses and commonly treated disease groups. ICF was calculated using formula [32]:

(3) $$\mathrm{ICF}\;=\;\frac{N_{\mathrm{ur}}\;-\;N_{t}}{N_{\mathrm{ur}}\;-1}$$

where N_ur_ represents the number of use-reports for a particular ailment category, and N_t_ is the number of taxa used for that category. The ICF value ranges from 0 to 1, where values close to 1 indicate a high level of agreement among informants (i.e., relatively few species are used by many people for a particular ailment), while values close to 0 suggest low consensus and a more diverse selection of plants used for the same condition.

## 3. Results

### 3.1. Diversity and Composition of Wild Edible Fruits

**Figure 2:**
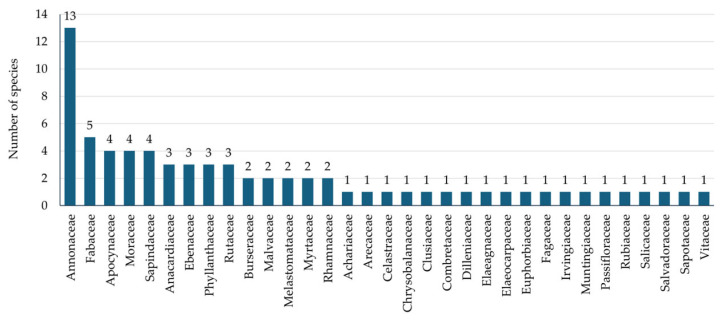
Bar chart showing the number of wild edible fruit species recorded in each plant family in Kut Chum District, Yasothon Province, Thailand.

**Figure 3:**
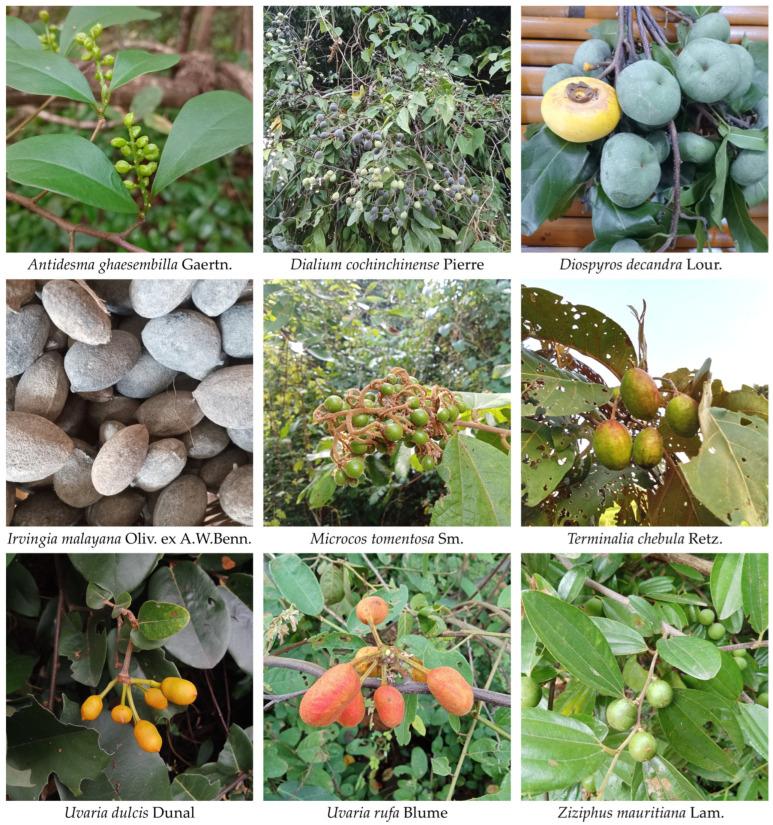
Representative wild edible fruits recorded in the study area of Kut Chum District, Yasothon Province, Thailand. Photos by Tammanoon Jitpromma.

A total of 71 wild edible fruit species, representing 59 genera and 33 plant families, were recorded in Kut Chum District, Yasothon Province (Figure 2 and Figure 3; Table 2). The documented diversity demonstrates the broad taxonomic range of species contributing to local food systems and traditional knowledge associated with wild fruit resources. The family Annonaceae was the most species-rich group in this dataset, comprising 13 species, indicating its prominent representation among wild edible fruit resources documented in the study area. This was followed by Fabaceae with five species. Several families, including Apocynaceae, Moraceae, and Sapindaceae, were represented by four species each, highlighting their consistent contribution to fruit-based plant use. Moderately represented families included Anacardiaceae, Ebenaceae, Phyllanthaceae, and Rutaceae, each contributing three species. Families with two species each comprised Burseraceae, Malvaceae, Melastomataceae, Myrtaceae, and Rhamnaceae. The remaining 19 families were represented by a single species, including Achariaceae, Arecaceae, Celastraceae, Chrysobalanaceae, Clusiaceae, Combretaceae, Dilleniaceae, Elaeagnaceae, Elaeocarpaceae, Euphorbiaceae, Fagaceae, Irvingiaceae, Muntingiaceae, Passifloraceae, Rubiaceae, Salicaceae, Salvadoraceae, Sapotaceae, and Vitaceae.

Regarding distribution status in Thailand, most species were native (66 species; 92.96%), while only a small proportion were introduced (5 species; 7.04%).

In terms of growth habit, trees were the dominant life form, accounting for 44 species (61.97%), followed by climbers (14 species; 19.72%) and shrubs (13 species; 18.31%).

### 3.2. Utilization of Wild Edible Fruits

#### 3.2.1. Wild Edible Fruits Used as Beverages

Although most wild edible fruits documented in the study area are primarily consumed fresh when ripe, a small number of species are occasionally used in the preparation of traditional beverages. In Kut Chum District, three species were reported to be utilized for this purpose: *Antidesma ghaesembilla*, *Syzygium cumini*, and *Ziziphus oenopolia* (Table 2).

The fruits of these species are generally eaten directly when ripe; however, local informants indicated that they may also be processed into simple drinks. Typically, the ripe fruits are crushed and mixed with drinking water at ambient temperature, and sometimes lightly sweetened to produce refreshing beverages. In some cases, the fruits may also be soaked in room-temperature water or briefly boiled in hot water to extract their flavor prior to consumption.

The preparation of beverages from these wild fruits represents an alternative form of utilization within local dietary practices. Although such uses are less common compared with direct fresh consumption, they demonstrate the versatility of wild edible fruits and their integration into everyday food traditions in the study area.

#### 3.2.2. Wild Edible Fruits Used as Fruit

Wild edible fruits constitute the largest utilization category documented in the study area. A total of 60 species were reported by local informants to be consumed primarily as fruits (Table 2). Most of these species are eaten directly when ripe and are commonly collected from forests, forest edges, fallow lands, and agricultural landscapes as seasonal snacks or supplementary food sources.

The majority of species (49 species) are consumed mainly when the fruits are fully ripe, as ripening generally enhances sweetness, reduces astringency, and improves palatability. Examples include *Alyxia schlechteri*, *Ampelocissus martini*, *Antidesma ghaesembilla*, *Artabotrys spinosus*, *Artocarpus lacucha*, *Azima sarmentosa*, *Buchanania lanzan*, *Calamus caesius*, *Dialium cochinchinense*, *Diospyros decandra*, *Flacourtia indica*, *Garcinia cowa*, *Lepisanthes rubiginosa*, *Muntingia calabura*, *Nephelium hypoleucum*, *Passiflora foetida*, *Pithecellobium dulce*, *Schleichera oleosa*, *Syzygium cumini*, *Uvaria dulcis*, *Willughbeia edulis*, and *Ziziphus mauritiana*, among many others.

A smaller group of species (eight species) is typically consumed at the unripe stage, often due to their sour or slightly astringent taste, which is preferred for direct consumption or for inclusion in traditional dishes. These species include *Acronychia pedunculata*, *Canarium subulatum*, *Feroniella lucida*, *Finlaysonia pierrei*, *Hymenocardia punctata*, *Irvingia malayana*, *Phyllanthus emblica*, and *Terminalia chebula*.

In addition, three species are consumed at both unripe and ripe stages, depending on taste preferences and availability. These include *Ficus hispida*, *Ficus racemosa*, and *Mangifera caloneura*.

#### 3.2.3. Wild Edible Fruits Used as Medicine

A total of 27 wild edible fruit species were documented as being utilized by local communities in Kut Chum District as both food and traditional medicinal resources (Table 2). These species are generally consumed as fruits in fresh or prepared forms while also being recognized for their health-related benefits within local knowledge systems. The dual role of these plants illustrates how edible fruits are integrated into everyday diets and traditional health practices.

Several species were reported to have overlapping uses between fruit consumption and medicinal applications. For instance, *Ficus hispida*, *Ficus racemosa*, *Irvingia malayana*, and *Phyllanthus emblica* are commonly eaten as fruits while also being regarded as plants with medicinal properties. Similarly, species such as *Acronychia pedunculata*, *Ampelocissus martini*, *Canthium berberidifolium*, *Hydnocarpus castaneus*, *Lepisanthes rubiginosa*, *Limonia acidissima*, and *Microcos tomentosa* are recognized both as edible fruits and as ingredients in traditional health-related practices.

In addition, several species belonging to the family Annonaceae, including *Huberantha cerasoides*, *Polyalthia debilis*, *Polyalthia evecta*, *Uvaria dulcis*, *Uvaria ferruginea*, *Uvaria siamensis*, and *Xylopia vielana*, were also recorded as edible fruits with medicinal roles. Other species documented in this category include *Buchanania siamensis*, *Cananga brandisiana*, *Diospyros filipendula*, *Goniothalamus laoticus*, *Grewia hirsuta*, *Melastoma malabathricum*, *Memecylon edule*, *Streblus asper*, and *Urceola polymorpha*.

#### 3.2.4. Wild Edible Fruits Used as Food Ingredient or Culinary Additive

Only one species was documented as being used as a food ingredient or culinary additive in the study area (Table 2). *Urceola polymorpha* is primarily consumed as a wild fruit; however, local informants also reported that its fruits are occasionally used as a souring agent in traditional dishes. The fruits are added to local soups to enhance acidity and flavor, particularly in dishes such as fish soup and chicken soup. This practice reflects the use of certain wild fruits not only as direct food sources but also as minor culinary ingredients that contribute to the characteristic taste of local cuisine.

### 3.3. Cultural Importance of Wild Edible Fruits

The Cultural Importance Index (CI) was calculated to evaluate the relative significance of wild edible fruit species used by local communities in Kut Chum District. The total CI values ranged from 0.133 to 0.867 among the documented species (Table 2 and Table S1). *Irvingia malayana* showed the highest CI value (0.867), followed by *Phyllanthus emblica* (0.833), *Ampelocissus martini* (0.767), *Syzygium cumini* (0.667), and *Schleichera oleosa* (0.650).

When analyzed by use categories, the fruit category had the highest cumulative CI value (23.067), followed by the medicinal category (5.283). The beverage category had a cumulative CI value of 0.217, while the food ingredient category showed a value of 0.317.

### 3.4. Ethnomedicine of Wild Edible Fruits

#### 3.4.1. Condition of Plant Material and Routes of Administration

**Figure 4:**
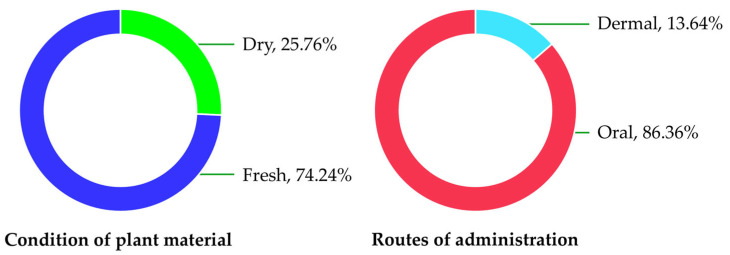
Proportion of condition of plant material and routes of administration.

The condition of plant materials used in the preparation of traditional remedies included both fresh and dried forms (Figure 4, Table 3). Most plant materials were used in fresh condition (74.24%), while 25.76% were utilized in dried form. Regarding the routes of administration, most remedies were taken orally (86.36%), whereas 13.64% were applied dermally.

#### 3.4.2. Plant Parts of Wild Edible Fruits Used as Medicine

**Figure 5:**
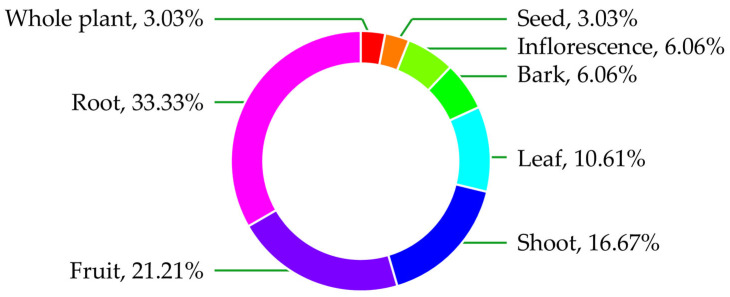
Proportional distribution of plant parts used in the preparation of medicinal remedies from wild edible fruits.

The plant parts used in the preparation of medicinal remedies from wild edible fruits varied among species (Figure 5). The root was the most frequently used part, accounting for 33.33% of the total uses. This was followed by fruits at 21.21% and shoots at 16.67%. Leaves contributed 10.61% of the reported uses. Bark and inflorescences were each used in 6.06% of cases, while seeds and whole plants were the least used, each representing 3.03% of the total.

#### 3.4.3. Fidelity Level (%FL) of Wild Edible Fruits Used as Medicine

The Fidelity Level (%FL) was applied to assess the relative importance of wild edible fruit species for specific therapeutic uses based on informant consensus (Table 3). The results revealed considerable variation in FL values among the documented species, reflecting differences in the level of agreement regarding their medicinal applications.

Several species exhibited very high FL values (100%), indicating strong consensus among informants for particular uses. These included *Acronychia pedunculata*, consistently used for treating fungal skin infections; *Grewia hirsuta*, commonly applied for burns and scald injuries; and *Xylopia vielana*, used to support cardiovascular health.

Moderately high FL values were also observed in several species. For example, *Canthium berberidifolium* showed a high FL (82.35%) for the treatment of abdominal abscesses, while *Ficus racemosa* and *Uvaria ferruginea* exhibited FL values of 70.00% for fever reduction and blood nourishment, respectively. Similarly, *Polyalthia debilis* (62.50%) was primarily used as an antipyretic, and *Lepisanthes rubiginosa* (50.00%) was commonly used to treat diarrhea.

Some species demonstrated multiple therapeutic uses with varying FL values, indicating broader medicinal roles but lower consensus for specific applications. For instance, *Ampelocissus martini* was used for fever reduction (56.25%), menstrual regulation (18.75%), and general antipyretic purposes (25.00%). Likewise, *Irvingia malayana* showed diversified uses, including joint strengthening (35.29%), laxative effects (23.53%), cough relief (23.53%), and anthelmintic treatment (17.65%). *Phyllanthus emblica* also displayed multiple uses with relatively lower FL values across different therapeutic categories.

#### 3.4.4. Informant Consensus Factor (ICF) of Wild Edible Fruits Used as Medicine

The Informant Consensus Factor (ICF) was calculated to evaluate the degree of agreement among informants regarding the use of wild edible fruits for different therapeutic categories (Table 4). The results showed generally high ICF values across all categories, ranging from 0.788 to 1.000, indicating strong consistency in traditional knowledge and plant use among the local community.

The highest ICF values (1.000) were recorded in the categories of reproductive disorders and poisoning and toxicology, suggesting complete agreement among informants, although these categories were represented by relatively few use-reports and species. High ICF values were also observed in central nervous system disorders (0.929), indicating a strong consensus for a limited number of species used to treat related conditions.

Categories with the highest number of use-reports included gastrointestinal disorders (N_ur_ = 82, ICF = 0.840) and infection, parasite and immune disorders (N_ur_ = 77, ICF = 0.842), reflecting both high usage frequency and substantial agreement among informants. Similarly, skin disorders (ICF = 0.806), musculoskeletal disorders (ICF = 0.826), and respiratory disorders (ICF = 0.789) demonstrated relatively high consensus values, indicating the importance of wild edible fruits in treating common health conditions.

Moderate to high ICF values were also found in general tonic (0.833), cardiological disorders (0.833), and obstetrics, gynaecology and urinary disorders (0.788), suggesting that these plant species play supportive roles in maintaining health and treating specific ailments within the community. Overall, the high ICF values highlight a well-established and shared body of ethnomedicinal knowledge related to wild edible fruits in the study area. It is noteworthy that some therapeutic categories exhibited high ICF values but were associated with relatively low %FL values for individual species. This pattern suggests that while there is strong agreement among informants regarding the treatment of specific ailment categories, multiple plant species are used interchangeably, resulting in lower specificity for any single species.

### 3.5. Medicinal Properties of Wild Edible Fruits Reported in Previous Studies

A total of 44 wild edible fruit species documented in the present study were not reported as medicinal plants within the study area. However, these species have been widely recorded for their therapeutic uses in other regions across northeastern Thailand (Table 5). This finding highlights the variability of ethnomedicinal knowledge and the context-dependent use of plant resources among different local communities.

Previous studies have shown that many of these species possess diverse medicinal properties and are used to treat a broad range of health conditions. The reported therapeutic applications include gastrointestinal disorders (e.g., *Dialium cochinchinense*, *Dillenia ovata*, *Salacia chinensis*), infections and immune-related diseases (e.g., *Artabotrys spinosus*, *Syzygium cumini*), and musculoskeletal disorders (e.g., *Sindora siamensis*, *Xylia xylocarpa*). Several species are also used in the treatment of fever and as antipyretics, such as *Diospyros decandra*, *Finlaysonia pierrei*, and *Protium serratum*.

In addition, certain species have been reported for more specific or specialized medicinal uses. For instance, *Azima sarmentosa* and *Buchanania lanzan* have been associated with cancer treatment, while *Ziziphus oenopolia* is traditionally used for managing diabetes and urinary disorders. Some species, such as *Muntingia calabura*, are recognized for their anti-inflammatory and antiseptic properties, whereas *Terminalia chebula* and *Passiflora foetida* are used for multiple conditions, including digestive, respiratory, and cardiovascular disorders.

## 4. Discussion

### 4.1. Diversity and Ethnobotanical Significance of Wild Edible Fruits

The documentation of 71 wild edible fruit species across 33 families in Kut Chum District reflects a remarkably high level of plant diversity embedded within local food systems. This richness is comparable to, and in some cases exceeds, findings from other ethnobotanical studies in both regional and global contexts. For instance, a previous study conducted in Roi Et Province, northeastern Thailand, recorded 68 wild edible fruit species [34], indicating a similar level of diversity within the same biogeographical region. Comparable levels of species richness have also been reported in Southeast Asia, such as in Bengkulu, Indonesia (73 species) [42] and Pesisir Selatan, West Sumatra, Indonesia (75 species) [43], where diverse forest ecosystems and strong traditional knowledge systems contribute to the extensive use of wild fruits.

In contrast, lower species richness has been documented in other regions, including studies among the Maale and Ari ethnic communities in southern Ethiopia (52 species) [44], Tabora region in Western Tanzania (27 species) [45], and in Karnataka, India (25 species) [46]. These differences may reflect variations in ecological conditions, forest availability, cultural practices, and the degree of dependence on wild plant resources among local communities.

The dominance of the family Annonaceae in the present study is particularly noteworthy and aligns with previous research highlighting its ecological adaptability and importance as a source of edible and medicinal species in tropical regions [34,44]. Overall, the relatively high diversity recorded in Kut Chum District underscores the rich ethnobotanical knowledge of local communities and the significant role of wild edible fruits in supporting both dietary diversity and cultural heritage.

The predominance of tree species (over 60%) highlights the fundamental role of arboreal plants in sustaining local food systems and supporting sustainable diets. Trees serve as long-term and reliable sources of wild edible fruits, contributing not only to food availability but also to nutritional diversity, particularly through the provision of vitamins, minerals, and other essential micronutrients [47]. In addition, tree-based resources are closely linked to cultural practices and traditional ecological knowledge, reinforcing their importance beyond subsistence [48]. From an environmental perspective, trees contribute to ecosystem stability, biodiversity conservation, and resilience within complex landscapes [49,50]. Collectively, these roles underscore the significance of tree species as key components in maintaining food security, supporting sustainable livelihoods, and preserving biocultural diversity.

The high proportion of native species (over 90%) indicates that local knowledge systems are deeply embedded in indigenous flora, reflecting long-term interactions between human communities and their surrounding ecosystems. This pattern is consistent with the concept of Indigenous Knowledge (IK), which represents cumulative, place-based knowledge developed through generations of observation, experimentation, and close engagement with local biodiversity [51]. Such knowledge systems contribute not only to the identification and utilization of wild edible fruits but also to a deeper understanding of ecological processes, species behavior, and seasonal dynamics [52]. The strong reliance on native species therefore underscores the adaptive and context-specific nature of local ethnobotanical knowledge, highlighting its value as a complementary framework to scientific research in advancing sustainable resource management and conservation practices [53].

### 4.2. Role of Wild Edible Fruits in Food Security

Wild edible fruits in Kut Chum District play a crucial role in enhancing local food security, particularly as seasonal and supplementary food resources. The predominance of fresh fruit consumption (60 species) highlights their accessibility and importance as immediate sources of energy and nutrients, especially during periods of agricultural scarcity or economic constraint [54]. Beyond their role as supplementary foods, wild edible fruits also contribute significantly to nutritional security by providing essential micronutrients, antioxidants, and bioactive compounds that support overall health and well-being [55].

In the context of increasing environmental and climatic uncertainties, such resources become even more important. Wild edible fruits represent naturally available and resilient food sources that can help buffer communities against fluctuations in agricultural production and food supply. Their ability to grow across diverse landscapes with minimal management further enhances their value as sustainable dietary components. Thus, these fruits function not only as fallback foods but also as vital elements of adaptive food systems, contributing to long-term food and nutrition security under changing environmental conditions [56]. Within this adaptive framework, species such as *Irvingia malayana*, *Phyllanthus emblica*, and *Syzygium cumini*, which exhibit high Cultural Importance Index (CI) values, further illustrate their central role in local diets. Their frequent use reflects not only availability but also strong cultural preference and perceived nutritional or health benefits, consistent with findings from other Southeast Asian contexts where culturally important fruit species contribute to the resilience of traditional food systems [57].

Moreover, the use of certain unripe fruits for sour taste in traditional diets reflects a nuanced understanding of flavor diversity and nutritional balance. Although only one species (*Urceola polymorpha*) was explicitly recorded as a culinary additive, this likely underrepresents the broader role of wild fruits in local gastronomy, as many uses may be context-specific or underreported [58].

### 4.3. Ethnomedicinal Knowledge and Health Applications

The integration of food and medicine is a defining feature of traditional knowledge systems in Kut Chum District. The documentation of 27 species used both as food and medicine underscores the concept of “food–medicine continuum,” widely recognized in ethnobotanical literature. Similar overlaps have been reported in studies across Thailand and other parts of Asia, where edible plants are routinely employed for preventive and therapeutic purposes [59,60,61].

The predominance of oral administration (86.36%) reflects the central role of dietary intake in traditional healing practices, while the use of fresh plant materials highlights the importance of accessibility and perceived efficacy [62]. High FL values for specific species, such as *Acronychia pedunculata* (dermatological infections) and *Grewia hirsuta* (burn treatment), indicate strong consensus and potential pharmacological relevance. Such high-consensus species are often considered prime candidates for further phytochemical and pharmacological investigation [63].

The high ICF values across multiple therapeutic categories (0.788–1.000) further demonstrate a well-established and shared body of ethnomedicinal knowledge. Particularly, the high consensus in gastrointestinal and infectious disease categories reflects the prevalence of these health conditions and the reliance on plant-based remedies for their management. Comparable ICF patterns have been observed in ethnomedicinal studies in rural Thailand, suggesting a consistent reliance on plant resources for primary healthcare [64].

### 4.4. Knowledge Variation and Cultural Transmission

An important finding of this study is that 44 species not used medicinally in the study area have been documented as medicinal plants in other parts of northeastern Thailand. This variation reflects the flexible and context-specific nature of ethnobotanical knowledge systems. Plant use is not uniform across regions but is shaped by local ecological conditions, cultural preferences, and historically developed knowledge systems within each community [65,66]. In this study area, these species are not recognized as medicinal plants in any form, suggesting that their exclusion from local medicinal practices is more likely related to differences in knowledge transmission, cultural selection, and perceived efficacy rather than the specific plant parts used.

In addition to environmental factors, ongoing socio-economic changes also influence how traditional knowledge is maintained and transmitted. Shifts toward modernization, changes in livelihood strategies, and improved access to formal education have altered traditional knowledge pathways. As younger generations increasingly relocate to urban centers for education and employment, opportunities for learning and practicing traditional plant use within communities may decline [67,68].

At the same time, increased population mobility has introduced new cultural interactions at the local level. The presence of migrants, temporary workers, and visitors can enrich local knowledge systems by bringing in alternative practices and perspectives [69]. However, these dynamics may also contribute to the gradual transformation or reduction of long-established traditional ecological knowledge. This evolving interplay between continuity and change highlights the importance of systematically documenting ethnobotanical knowledge before it is further modified or lost [70].

Such spatial variation underscores the importance of localized studies in capturing the full spectrum of ethnobotanical knowledge. It also suggests that some species may possess underutilized medicinal potential within the study area, representing opportunities for knowledge exchange and revitalization of traditional practices.

### 4.5. Conservation Implications

The high diversity of wild edible fruits documented in this study emphasizes the need for targeted and context-specific conservation strategies. The predominance of tree species suggests that ongoing deforestation, land-use change, and agricultural intensification could substantially reduce the availability of these resources, with direct consequences for both food security and local livelihoods [71,72]. Such pressures not only threaten plant diversity but may also disrupt the ecological foundations that sustain traditional foraging systems.

Effective conservation approaches should therefore integrate both ecological and socio-cultural dimensions. In particular, the role of culturally important species offers a valuable entry point for strengthening community engagement in conservation initiatives [73,74]. Species with high cultural importance—those that play significant roles in local diets, health practices, and cultural identity—are more likely to motivate local participation in resource management [75,76]. When communities perceive direct benefits or strong cultural connections to specific plant species, they are generally more willing to invest time and effort in conservation actions [77].

Building on this perspective, conservation strategies that prioritize culturally important species may enhance the success of community-based and co-management approaches. These species can function as focal points for conservation planning, helping to align biodiversity protection with local values and needs [78]. Moreover, safeguarding such species is critical not only for maintaining biological diversity but also for preserving traditional ecological knowledge (TEK) and ensuring the continuity of cultural practices associated with wild edible plants [79,80].

### 4.6. Potential for Future Pharmacological Research

The ethnomedicinal data presented in this study highlight significant potential for future drug discovery and development. Species with high FL and ICF values, as well as those with documented medicinal uses in other regions, represent promising candidates for further investigation.

For example, species such as *Phyllanthus emblica* and *Terminalia chebula* are already well-known in traditional medicine systems and have demonstrated pharmacological activities, including antioxidant, antimicrobial, and anti-inflammatory properties. Similarly, lesser-studied species identified in this study, particularly within the Annonaceae family, may harbor novel bioactive compounds.

The overlap between nutritional and medicinal uses also suggests that many of these fruits could be explored as functional foods or nutraceuticals. Integrating ethnobotanical knowledge with modern phytochemical and pharmacological research could therefore contribute to the development of sustainable and culturally relevant healthcare solutions.

However, several limitations should be considered when interpreting these findings. The ethnomedicinal data are based on knowledge reported by local informants at a specific point in time and may not fully capture temporal changes in plant use or knowledge dynamics. In addition, the study focused specifically on wild edible fruits, which may exclude other medicinal plant species used within the community. Furthermore, the observed patterns of use and cultural importance are context-dependent and may not be directly generalizable to other regions with different ecological and socio-cultural settings. The transmission of traditional knowledge, which largely occurs through informal and experience-based processes, may also be uneven across generations, particularly under the influence of modernization, migration, and changing livelihood strategies. This may result in partial knowledge retention or underreporting of certain uses. Future studies incorporating longitudinal data and comparative analyses across regions would provide a more comprehensive understanding of ethnobotanical knowledge systems and their applications.

## 5. Conclusions

This study documents a high diversity of wild edible fruit species (71 species across 33 families) in Kut Chum District, Yasothon Province, underscoring the richness of biocultural resources embedded within local food systems. The predominance of native and arboreal species highlights the strong ecological basis of these resources and their close association with forest and semi-natural landscapes. Wild edible fruits were shown to play multifunctional roles, contributing not only to dietary diversity and nutritional security but also to traditional healthcare practices, as reflected by their substantial representation in both food and medicinal use categories.

Quantitative ethnobotanical indices, including the Cultural Importance Index (CI), Fidelity Level (%FL), and Informant Consensus Factor (ICF), reveal a high degree of cultural relevance and shared knowledge among local informants. Species such as *Irvingia malayana*, *Phyllanthus emblica*, and *Syzygium cumini* emerged as key taxa within the local food system, indicating their potential as culturally important species with both nutritional and functional value. At the same time, the observed variation in medicinal plant use—where several species are recognized as medicinal in other regions but not locally—highlights the dynamic, context-dependent nature of ethnobotanical knowledge and the influence of socio-cultural change on knowledge transmission.

These findings have important implications for conservation and sustainable resource management. Integrating culturally important species into conservation planning may enhance community engagement and improve the effectiveness of co-management strategies. Protecting these species is not only essential for maintaining biodiversity but also for safeguarding traditional ecological knowledge and cultural continuity.

Overall, wild edible fruits represent a critical yet underutilized component of sustainable food systems. Their dual role in nutrition and health, combined with their resilience under changing environmental conditions, positions them as promising candidates for future research on functional foods, nutraceutical development, and climate-adaptive food strategies. Further interdisciplinary studies are needed to explore their phytochemical properties, nutritional profiles, and potential integration into broader food and health systems.

## Figures and Tables

**Table 1:** Demographic characteristics and geographic locations of informants in the study area, including sub-districts, GPS coordinates, gender, ethnicity, language, and religion.

Sub-District	GPS Coordinates		Gender		Ethnicity	Language	Religion
	Latitude (N, S)	Longitude (E, W)	Male	Female			
Huai Kaeng	16°02′01″ N	104°14′38″ E	3	3	Lao Isan	Isan language	Theravāda Buddhism
Kammaet	15°55′20″ N	104°21′11″ E	3	3	Lao Isan	Isan language	Theravāda Buddhism
Kham Nam Sang	16°05′36″ N	104°15′27″ E	4	4	Lao Isan	Isan language	Christianity, Theravāda Buddhism
Kut Chum	16°06′45″ N	104°19′59″ E	4	4	Lao Isan	Isan language	Christianity, Theravāda Buddhism
Na So	15°57′42″ N	104°19′44″ E	3	3	Lao Isan	Isan language	Theravāda Buddhism
Non Pueai	16°00′19″ N	104°22′37″ E	3	3	Lao Isan	Isan language	Theravāda Buddhism
Nong Mi	16°04′16″ N	104°14′04″ E	3	3	Lao Isan	Isan language	Theravāda Buddhism
Nong Nae	16°08′30″ N	104°14′24″ E	3	3	Lao Isan	Isan language	Christianity, Theravāda Buddhism
Phon Ngam	16°04′56″ N	104°11′38″ E	4	4	Lao Isan	Isan language	Theravāda Buddhism

**Table 2:** Documented wild edible fruit species in Kut Chum District, Yasothon Province, including family name, scientific name, vernacular names, distribution status in Thailand (DiT), growth habit (GH), utilization categories, use reports, cultural importance index (CI), and voucher numbers (VN).

Family	Scientific Name	Vernacular Name	DiT	GH	Utilization	Use-Reports				CI	VN
						BV	FT	MD	SP		
Achariaceae	*Hydnocarpus castaneus* Hook.f. & Thomson	Bak kabao	NT	Tree	FT	-	5	8	-	0.217	TJ-F0033
Anacardiaceae	*Buchanania lanzan* Spreng.	Bak muang hua maeng wan	NT	Tree	FT	-	23	-	-	0.383	TJ-F0008
Anacardiaceae	*Buchanania siamensis* Miq.	Bak huang sai	NT	Tree	MD	-	-	12	-	0.200	TJ-F0009
Anacardiaceae	*Mangifera caloneura* Kurz	Bak muang pa	NT	Tree	FT	-	32	-	-	0.533	TJ-F0040
Annonaceae	*Artabotrys spinosus* Craib	Bak nao nam	NT	Climber	FT	-	13	-	-	0.217	TJ-F0005
Annonaceae	*Cananga brandisiana* (Pierre) Saff.	Bak sakae saeng	NT	Tree	MD	-	-	11	-	0.183	TJ-F0012
Annonaceae	*Dasymaschalon lomentaceum* Finet & Gagnep.	Bak khoy ling	NT	Tree	FT	-	15	-	-	0.250	TJ-F0016
Annonaceae	*Goniothalamus laoticus* (Finet & Gagnep.) Bân	Bak khaolamdong	NT	Shrub	MD	-	-	10	-	0.167	TJ-F0030
Annonaceae	*Huberantha cerasoides* (Roxb.) Chaowasku	Bak ka chian	NT	Tree	MD	-	-	11	-	0.183	TJ-F0032
Annonaceae	*Polyalthia debilis* (Pierre) Finet & Gagnep.	Bak kluai tao	NT	Shrub	FT, MD	-	11	8	-	0.317	TJ-F0050
Annonaceae	*Polyalthia evecta* (Pierre) Finet & Gagnep.	Bak tong laeng	NT	Shrub	FT, MD	-	21	9	-	0.500	TJ-F0051
Annonaceae	*Uvaria dulcis* Dunal	Bak nomngua	NT	Climber	FT, MD	-	13	7	-	0.333	TJ-F0056
Annonaceae	*Uvaria ferruginea* Buch.-Ham. ex Hook.f. & Thomson	Bak nom maeopa	NT	Climber	FT, MD	-	12	11	-	0.383	TJ-F0044
Annonaceae	*Uvaria ferruginea* var. *cherrevensis* (Pierre ex Finet & Gagnep.) Meade & J.Parn.	Bak tit tang	NT	Climber	FT	-	16	-	-	0.267	TJ-F0058
Annonaceae	*Uvaria rufa* Blume	Bak phi phuan	NT	Climber	FT	-	38	-	-	0.633	TJ-F0059
Annonaceae	*Uvaria siamensis* (Scheff.) L.L.Zhou, Y.C.F.Su & R.M.K.Saunders	Bak lamduan	NT	Climber	MD	-	-	19	-	0.317	TJ-F0047
Annonaceae	*Xylopia vielana* Pierre	Bak kluai noi	NT	Tree	MD	-	-	18	-	0.300	TJ-F0004
Apocynaceae	*Alyxia schlechteri* H.Lév.	Bak tang tun	NT	Climber	FT	-	9	-	-	0.150	TJ-F0034
Apocynaceae	*Finlaysonia pierrei* (Costantin) Venter	Bak tam yan	NT	Climber	FT	-	27	-	-	0.450	TJ-F0048
Apocynaceae	*Urceola polymorpha* (Pierre ex Spire) D.J.Middleton & Livsh.	Bak som lom	NT	Climber	MD, SP	-	-	10	19	0.483	TJ-F0070
Apocynaceae	*Willughbeia edulis* Roxb.	Bak yang	NT	Climber	FT	-	35	-	-	0.583	TJ-F0071
Arecaceae	*Calamus caesius* Blume	Bak wai	NT	Tree	FT	-	21	-	-	0.350	TJ-F0014
Burseraceae	*Canarium subulatum* Guillaumin	Bak liam	NT	Tree	FT	-	27	-	-	0.450	TJ-F0001
Burseraceae	*Protium serratum* (Wall. ex Colebr.) Engl.	Bak faen	NT	Tree	FT	-	18	-	-	0.300	TJ-F0024
Celastraceae	*Salacia chinensis* L.	Bak ka kai	NT	Climber	FT	-	19	-	-	0.317	TJ-F0038
Chrysobalanaceae	*Parinari anamensis* Hance	Bak phok	NT	Tree	FT	-	17	-	-	0.283	TJ-F0028
Clusiaceae	*Garcinia cowa* Roxb. ex Choisy	Bak mong	NT	Tree	FT	-	16	-	-	0.267	TJ-F0007
Combretaceae	*Terminalia chebula* Retz.	Bak som mo	NT	Tree	FT	-	26	-	-	0.433	TJ-F0036
Dilleniaceae	*Dillenia ovata* Wall. ex Hook.f. & Thomson	Bak san	NT	Tree	FT	-	27	-	-	0.450	TJ-F0037
Ebenaceae	*Diospyros decandra* Lour.	Bak chan	NT	Tree	FT	-	36	-	-	0.600	TJ-F0045
Ebenaceae	*Diospyros filipendula* Pierre ex Lecomte	Bak khan chong	NT	Tree	MD	-	-	20	-	0.333	TJ-F0054
Ebenaceae	*Diospyros rhodocalyx* Kurz	Bak ko na	NT	Tree	FT	-	12	-	-	0.200	TJ-F0039
Elaeagnaceae	*Elaeagnus latifolia* L.	Bak lot	NT	Shrub	FT	-	11	-	-	0.183	TJ-F0003
Elaeocarpaceae	*Elaeocarpus hygrophilus* Kurz	Bak saeo	NT	Tree	FT	-	32	-	-	0.533	TJ-F0062
Euphorbiaceae	*Suregada multiflora* (A.Juss.) Baill.	Bak duk	NT	Shrub	FT	-	13	-	-	0.217	TJ-F0063
Fabaceae	*Cajanus cajan* (L.) Huth	Bak thua hae	IT	Shrub	FT	-	22	-	-	0.367	TJ-F0064
Fabaceae	*Dialium cochinchinense* Pierre	Bak kheng	NT	Tree	FT	-	29	-	-	0.483	TJ-F0065
Fabaceae	*Pithecellobium dulce* (Roxb.) Benth.	Bak kham pae	IT	Tree	FT	-	34	-	-	0.567	TJ-F0066
Fabaceae	*Sindora siamensis* Teijsm. ex Miq.	Bak tae	NT	Tree	FT	-	20	-	-	0.333	TJ-F0069
Fabaceae	*Xylia xylocarpa* (Roxb.) W.Theob.	Bak daeng	NT	Tree	FT	-	21	-	-	0.350	TJ-F0002
Fagaceae	*Castanopsis piriformis* Hickel & A.Camus	Bak ko	NT	Tree	FT	-	23	-	-	0.383	TJ-F0027
Irvingiaceae	*Irvingia malayana* Oliv. ex A.W.Benn.	Bak bok	NT	Tree	FT, MD	-	37	15	-	0.867	TJ-F0061
Malvaceae	*Grewia hirsuta* Vahl	Bak khao chi	NT	Shrub	MD	-		13	-	0.217	TJ-F0067
Malvaceae	*Microcos tomentosa* Sm.	Bak khom	NT	Tree	FT, MD	-	24	12	-	0.600	TJ-F0011
Melastomataceae	*Melastoma malabathricum* L.	Bak khlong khleng	NT	Shrub	MD	-	-	13	-	0.217	TJ-F0013
Melastomataceae	*Memecylon edule* Roxb.	Bak phlong mueat	NT	Tree	MD	-	-	10	-	0.167	TJ-F0052
Moraceae	*Artocarpus lacucha* Buch.-Ham.	Bak hat	NT	Tree	FT	-	37	-	-	0.617	TJ-F0053
Moraceae	*Ficus hispida* L.f.	Bak duea plong	NT	Tree	FT, MD	-	21	9	-	0.500	TJ-F0046
Moraceae	*Ficus racemosa* L.	Bak duea chumphon	NT	Tree	FT, MD	-	20	11	-	0.517	TJ-F0029
Moraceae	*Streblus asper* Lour.	Bak khoi	NT	Tree	FT	-	22	14	-	0.600	TJ-F0060
Muntingiaceae	*Muntingia calabura* L.	Bak takop	IT	Tree	FT	-	32	-	-	0.533	TJ-F0018
Myrtaceae	*Syzygium antisepticum* (Blume) Merr. & L.M.Perry	Bak mek	NT	Tree	FT	-	31	-	-	0.517	TJ-F0019
Myrtaceae	*Syzygium cumini* (L.) Skeels	Bak wa	NT	Tree	BV, FT	5	35	-	-	0.667	TJ-F0020
Passifloraceae	*Passiflora foetida* L.	Bak krathok rok pa	IT	Climber	FT	-	16	-	-	0.267	TJ-F0021
Phyllanthaceae	*Antidesma ghaesembilla* Gaertn.	Bak mao khi nok	NT	Shrub	BV, FT	6	32	-	-	0.633	TJ-F0022
Phyllanthaceae	*Hymenocardia punctata* Wall. ex Lindl.	Bak hu ling	NT	Shrub	FT	-	18	-	-	0.300	TJ-F0023
Phyllanthaceae	*Phyllanthus emblica* L.	Bak kham pom	NT	Tree	FT, MD	-	35	15	-	0.833	TJ-F0057
Rhamnaceae	*Ziziphus mauritiana* Lam.	Bak than	NT	Tree	FT	-	33	-	-	0.550	TJ-F0010
Rhamnaceae	*Ziziphus oenopolia* (L.) Mill.	Bak lep maeo	NT	Climber	BV, FT	2	36	-	-	0.633	TJ-F0017
Rubiaceae	*Canthium berberidifolium* E.T.Geddes	Bak ngiang duk	NT	Shrub	MD	-	6	10	-	0.267	TJ-F0049
Rutaceae	*Acronychia pedunculata* (L.) Miq.	Bak yom pa	NT	Tree	MD	-	9	12	-	0.350	TJ-F0055
Rutaceae	*Feroniella lucida* (Scheff.) Swingle	Bak sang	NT	Tree	FT	-	8	-	-	0.133	TJ-F0068
Rutaceae	*Limonia acidissima* L.	Bak khwit	IT	Tree	FT	-	15	6	-	0.350	TJ-F0015
Salicaceae	*Flacourtia indica* (Burm.f.) Merr.	Bak ben	NT	Shrub	FT	-	37	-	-	0.617	TJ-F0035
Salvadoraceae	*Azima sarmentosa* (Blume) Benth. & Hook.f.	Bak haet	NT	Shrub	FT	-	12	-	-	0.200	TJ-F0031
Sapindaceae	*Lepisanthes rubiginosa* (Roxb.) Leenh.	Bak huat kha	NT	Tree	FT, MD	-	26	8	-	0.567	TJ-F0043
Sapindaceae	*Lepisanthes senegalensis* (Poir.) Leenh.	Bak ma wo	NT	Tree	FT	-	19	-	-	0.317	TJ-F0041
Sapindaceae	*Nephelium hypoleucum* Kurz	Bak ngao	NT	Tree	FT	-	37	-	-	0.617	TJ-F0042
Sapindaceae	*Schleichera oleosa* (Lour.) Oken	Bak kho	NT	Tree	FT	-	39	-	-	0.650	TJ-F0006
Sapotaceae	*Madhuca thorelii* (Pierre ex Dubard) H.J.Lam	Bak dueai kai	NT	Tree	FT	-	22	-	-	0.367	TJ-F0025
Vitaceae	*Ampelocissus martini* Planch.	Bak koi	NT	Climber	FT, MD	-	31	15	-	0.767	TJ-F0026

Abbreviation. Distribution status in Thailand: NT (native), IT (introduced). Utilization: BV (beverages), FT (fruit), MD (medicine), SP (food ingredient or culinary additive).

**Table 3:** Ethnomedicinal uses of recorded plant species, including scientific name, fidelity level (FL), used parts (UP), condition of plant material (CoP), preparation methods, routes of administration (RoA), traditional applications, and therapeutic categories.

Scientific Name	FL	UP	CoP	Preparation	RoA	Traditional Application	Therapeutic Categories
*Acronychia pedunculata* (L.) Miq.	100.00	BK	Fresh	Pounded into a fine paste and applied to the affected skin area.	Dermal	Traditionally used to relieve itching, ringworm, and other fungal skin infections.	Skin Disorders
*Ampelocissus martini* Planch.	56.25	LV	Fresh	Prepared as a decoction; the filtrate is taken orally.	Oral	Traditionally used to reduce fever.	Infection, Parasite and Immune Disorders
	18.75	SH	Fresh	Prepared as a decoction; the filtrate is taken orally.	Oral	Traditionally used to cleanse menstrual blood and stimulate menstrual flow.	Obstetrics, Gynaecology and Urinary Disorders
	25.00	RT	Fresh	Rubbed with water and the extract is drunk.	Oral	Traditionally used to reduce fever.	Infection, Parasite and Immune Disorders
*Buchanania siamensis* Miq.	42.86	BK	Fresh	Boiled with salt and used as a mouth rinse.	Oral	Traditionally used to treat gingivitis and oral ulcers.	Gastrointestinal Disorders
	57.14	BK	Fresh	Boiled and applied to the affected area.	Dermal	Traditionally used to treat herpes infections.	Infection, Parasite and Immune Disorders
*Cananga brandisiana* (Pierre) Saff.	46.15	LV	Fresh	Leaves are burned and the smoke is used to fumigate the affected area.	Dermal	Traditionally used to treat chronic skin parasites and persistent wounds.	Skin Disorders
	53.85	WP	Fresh	The whole plant is pounded and the juice is extracted and drunk.	Oral	Traditionally used to reduce fever.	Infection, Parasite and Immune Disorders
*Canthium berberidifolium* E.T.Geddes	82.35	RT	Fresh	Prepared as a decoction; the filtrate is taken orally.	Oral	Traditionally used to treat abdominal abscesses.	Gastrointestinal Disorders
	17.65	RT	Fresh	Prepared as a decoction; the filtrate is taken orally.	Oral	Traditionally used to relieve bloating.	Gastrointestinal Disorders
*Diospyros filipendula* Pierre ex Lecomte	27.27	RT	Fresh	Prepared as a decoction; the filtrate is taken orally.	Oral	Traditionally used to treat abdominal pain and dysentery.	Gastrointestinal Disorders
	36.36	RT	Fresh	Prepared as a decoction; the filtrate is taken orally.	Oral	Traditionally used to reduce fever.	Infection, Parasite and Immune Disorders
	36.36	RT	Fresh	Boiled and used for bathing.	Dermal	Traditionally used to restore strength in postpartum women unable to undergo traditional heat therapy.	Obstetrics, Gynaecology and Urinary Disorders
*Ficus hispida* L.f.	45.45	FT	Fresh	Eaten fresh.	Oral	Traditionally used to reduce fever.	Infection, Parasite and Immune Disorders
	54.55	FT	Fresh	Eaten fresh.	Oral	Traditionally used to treat diarrhea.	Gastrointestinal Disorders
*Ficus racemosa* L.	70.00	RT	Dry	Prepared as a decoction; the filtrate is taken orally.	Oral	Traditionally used to reduce fever.	Infection, Parasite and Immune Disorders
	30.00	FT	Fresh	Eaten fresh.	Oral	Traditionally used to alleviate internal heat.	Infection, Parasite and Immune Disorders
*Goniothalamus laoticus* (Finet & Gagnep.) Bân	42.86	SH	Fresh	Prepared as a decoction; the filtrate is taken orally.	Oral	Traditionally used to promote uterine recovery and enhance lactation in postpartum women.	Obstetrics, Gynaecology and Urinary Disorders
	57.14	SH	Fresh	Prepared as a decoction; the filtrate is taken orally.	Oral	Traditionally used as a general body tonic.	General Tonic
*Grewia hirsuta* Vahl	100.00	LV	Fresh	Fresh leaves are crushed and applied as a poultice to the wound.	Dermal	Traditionally used to treat burns and scald injuries.	Skin Disorders
*Huberantha cerasoides* (Roxb.) Chaowasku	33.33	SH	Fresh	Prepared as a decoction; the filtrate is taken orally.	Oral	Traditionally used to relieve muscle pain and body aches.	Musculoskeletal Disorders
	40.00	RT	Fresh	Prepared as a decoction; the filtrate is taken orally.	Oral	Traditionally used to enhance male sexual vitality.	Reproductive Disorders
	26.67	LV	Fresh	Pounded and applied as a poultice to the affected area.	Dermal	Traditionally used to treat inflamed wounds.	Skin Disorders
*Hydnocarpus castaneus* Hook.f. & Thomson	38.46	RT	Fresh	Prepared as a decoction; the filtrate is taken orally.	Oral	Traditionally used for detoxification.	Poisoning and Toxicology
	38.46	RT	Fresh	Prepared as a decoction; the filtrate is taken orally.	Oral	Traditionally used to reduce phlegm.	Infection, Parasite and Immune Disorders
	23.08	RT	Fresh	Pounded, mixed with alcohol, and applied topically to the skin.	Dermal	Traditionally used to treat ringworm and other fungal skin infections.	Skin Disorders
*Irvingia malayana* Oliv. ex A.W.Benn.	17.65	FT	Fresh	Prepared as a decoction; the filtrate is taken orally.	Oral	Traditionally used to expel intestinal worms in children.	Infection, Parasite and Immune Disorders
	23.53	FT	Fresh	Prepared as a decoction; the filtrate is taken orally.	Oral	Traditionally used as a laxative to relieve constipation.	Gastrointestinal Disorders
	35.29	SD	Fresh	Eaten fresh.	Oral	Traditionally used to strengthen joints, bones, and tendons.	Musculoskeletal Disorders
	23.53	SH	Fresh	Prepared as a decoction; the filtrate is taken orally.	Oral	Traditionally used to relieve cough.	Respiratory Disorders
*Lepisanthes rubiginosa* (Roxb.) Leenh.	28.57	SD	Dry	Prepared as a decoction; the filtrate is taken orally.	Oral	Traditionally used as an anthelmintic.	Infection, Parasite and Immune Disorders
	50.00	FT	Fresh	Eaten fresh.	Oral	Traditionally used to treat diarrhea.	Gastrointestinal Disorders
	21.43	LV	Fresh	Boiled and used for bathing.	Dermal	Traditionally used to relieve skin rashes, itching, and other skin conditions.	Skin Disorders
*Limonia acidissima* L.	44.44	LV	Fresh	Prepared as a decoction; the filtrate is taken orally.	Oral	Traditionally used to treat diarrhea.	Gastrointestinal Disorders
	55.56	FT	Fresh	Eaten fresh.	Oral	Traditionally used to expel intestinal worms.	Infection, Parasite and Immune Disorders
*Melastoma malabathricum* L.	25.00	RT	Fresh	Prepared as a decoction; the filtrate is taken orally.	Oral	Traditionally used to strengthen the body.	General Tonic
	29.17	IF	Dry	Prepared as a decoction; the filtrate is taken orally.	Oral	Traditionally used as a sedative.	Central Nervous System Disorders
	45.83	LV	Fresh	Prepared as a decoction; the filtrate is taken orally.	Oral	Traditionally used to treat diarrhea and dysentery.	Gastrointestinal Disorders
*Memecylon edule* Roxb.	45.45	RT	Fresh	Prepared as a decoction; the filtrate is taken orally.	Oral	Traditionally used to treat gastric disorders.	Gastrointestinal Disorders
	54.55	SH	Fresh	Prepared as a decoction; the filtrate is taken orally.	Oral	Traditionally used to relieve asthma symptoms.	Respiratory Disorders
*Microcos tomentosa* Sm.	21.43	SH	Fresh	Prepared as a decoction; the filtrate is taken orally.	Oral	Traditionally used to relieve asthma symptoms.	Respiratory Disorders
	21.43	BK	Fresh	Prepared as a decoction; the filtrate is taken orally.	Oral	Traditionally used to nourish women’s blood.	Obstetrics, Gynaecology and Urinary Disorders
	57.14	FT	Fresh	Eaten fresh.	Oral	Traditionally used as a laxative for constipation.	Gastrointestinal Disorders
*Phyllanthus emblica* L.	13.33	FT	Dry	Eaten directly.	Oral	Traditionally used to reduce phlegm.	Infection, Parasite and Immune Disorders
	20.00	FT	Dry	Eaten directly.	Oral	Traditionally used to aid digestion.	Gastrointestinal Disorders
	20.00	FT	Fresh	Eaten fresh.	Oral	Traditionally used to relieve cough.	Respiratory Disorders
	46.67	FT	Fresh	Eaten fresh.	Oral	Traditionally used to expel intestinal worms.	Infection, Parasite and Immune Disorders
*Polyalthia debilis* (Pierre) Finet & Gagnep.	37.50	RT	Dry	Prepared as a decoction; the filtrate is taken orally.	Oral	Traditionally used to stimulate menstruation.	Obstetrics, Gynaecology and Urinary Disorders
	62.50	RT	Dry	Prepared as a decoction; the filtrate is taken orally.	Oral	Traditionally used to reduce fever.	Infection, Parasite and Immune Disorders
*Polyalthia evecta* (Pierre) Finet & Gagnep.	26.67	RT	Dry	Prepared as a decoction; the filtrate is taken orally.	Oral	Traditionally used to treat gastric ulcers.	Gastrointestinal Disorders
	33.33	SH	Dry	Prepared as a decoction; the filtrate is taken orally.	Oral	Traditionally used to relieve muscle pain.	Musculoskeletal Disorders
	40.00	SH	Fresh	Prepared as a decoction; the filtrate is taken orally.	Oral	Traditionally used as a postpartum tonic.	Obstetrics, Gynaecology and Urinary Disorders
*Streblus asper* Lour.	42.86	RT	Fresh	Prepared as a decoction; the filtrate is taken orally.	Oral	Traditionally used to treat bone disorders, nerve pain, and lower back pain.	Musculoskeletal Disorders
	57.14	FT	Fresh	Eaten fresh.	Oral	Traditionally used to relieve cough.	Respiratory Disorders
*Urceola polymorpha* (Pierre ex Spire) D.J.Middleton & Livsh.	26.67	RT	Fresh	Prepared as a decoction; the filtrate is taken orally.	Oral	Traditionally used to treat abdominal pain, bloating, and digestive discomfort.	Gastrointestinal Disorders
	33.33	RT	Fresh	Prepared as a decoction; the filtrate is taken orally.	Oral	Traditionally used to relieve body aches and muscular pain.	Musculoskeletal Disorders
	40.00	WP	Fresh	Boiled and used for bathing.	Dermal	Traditionally used to relieve itchy skin.	Skin Disorders
*Uvaria dulcis* Dunal	41.67	RT	Dry	Prepared as a decoction; the filtrate is taken orally.	Oral	Traditionally used to promote lactation in postpartum women.	Obstetrics, Gynaecology and Urinary Disorders
	58.33	FT	Fresh	Eaten fresh.	Oral	Traditionally used as a general tonic.	General tonic
*Uvaria ferruginea* Buch.-Ham. ex Hook.f. & Thomson	70.00	RT	Dry	Prepared as a decoction; the filtrate is taken orally.	Oral	Traditionally used to nourish women’s blood.	Obstetrics, Gynaecology and Urinary Disorders
	30.00	RT	Dry	Prepared as a decoction; the filtrate is taken orally.	Oral	Traditionally used to treat disorders of the small intestine.	Gastrointestinal Disorders
*Uvaria siamensis* (Scheff.) L.L.Zhou, Y.C.F.Su & R.M.K.Saunders	18.75	SH	Dry	Prepared as a decoction; the filtrate is taken orally.	Oral	Traditionally used to support cardiovascular health.	Cardiological Disorders
	50.00	SH	Dry	Prepared as a decoction; the filtrate is taken orally.	Oral	Traditionally used to relieve dizziness associated with wind disorders.	Central Nervous System Disorders
	12.50	IF	Dry	Prepared as a decoction; the filtrate is taken orally.	Oral	Traditionally used to strengthen the body.	General Tonic
	18.75	IF	Dry	Prepared as a decoction; the filtrate is taken orally.	Oral	Traditionally used to reduce fever.	Infection, Parasite and Immune Disorders
*Xylopia vielana* Pierre	100.00	IF	Dry	Prepared as a decoction; the filtrate is taken orally.	Oral	Traditionally used to support cardiovascular health.	Cardiological Disorders

Abbreviation: Used part: BK (bark), FT (fruit), IF (inflorescence), LV (leaf), RT (root), SD (seed), SH (stem or shoot), WP (whole plant).

**Table 4:** Informant Consensus Factor (ICF) values across therapeutic categories of wild edible fruits used as medicine.

Therapeutic Categories	N_ur_	N_t_	ICF
Reproductive Disorders	6	1	1.000
Poisoning and Toxicology	5	1	1.000
Central Nervous System Disorders	15	2	0.929
Infection, Parasite and Immune Disorders	77	13	0.842
Gastrointestinal Disorders	82	14	0.840
General Tonic	19	4	0.833
Cardiological Disorders	7	2	0.833
Musculoskeletal Disorders	24	5	0.826
Skin Disorders	32	7	0.806
Respiratory Disorders	20	5	0.789
Obstetrics, Gynaecology and Urinary Disorders	34	8	0.788

**Table 5:** Medicinal uses of wild edible fruit species not utilized as medicine in the study area but reported in previous studies from northeastern Thailand.

Scientific Name	Traditional Uses	Reference
*Alyxia schlechteri* H.Lév.	Used to treat infections, parasitic infestations, and fever	[33]
*Antidesma ghaesembilla* Gaertn.	Used to treat musculoskeletal disorders, gynecological and urinary disorders, blood-related conditions, and fever	[34]
*Artabotrys spinosus* Craib	Used to treat infections, parasitic diseases, and immune-related disorders	[34]
*Artocarpus lacucha* Buch.-Ham.	Used to treat infections, intestinal worms, oral ulcers, hypertension, respiratory disorders (cough and cold), flatulence, and musculoskeletal pain	[33,34,35,36,37]
*Azima sarmentosa* (Blume) Benth. & Hook.f.	Used in the treatment or prevention of cancer	[38]
*Buchanania lanzan* Spreng.	Used to treat cancer, skin diseases, body pain, diarrhea, and gastric ulcers	[39]
*Cajanus cajan* (L.) Huth	Used to treat musculoskeletal disorders, gynecological and urinary disorders, blood-related conditions, and fever	[34]
*Calamus caesius* Blume	Used to treat blood-related conditions and fever	[34]
*Canarium subulatum* Guillaumin	Used to treat mouth ulcers, fever, respiratory disorders, eye infections, and skin diseases	[35,37,39]
*Castanopsis piriformis* Hickel & A.Camus	Used for wound healing and treatment of blood-related conditions	[33,34]
*Dasymaschalon lomentaceum* Finet & Gagnep.	Used to treat musculoskeletal disorders	[34]
*Dialium cochinchinense* Pierre	Used to treat fever and gastrointestinal disorders	[34,35]
*Dillenia ovata* Wall. ex Hook.f. & Thomson	Used to treat fever and gastrointestinal disorders	[34]
*Diospyros decandra* Lour.	Used to treat fever	[34]
*Diospyros rhodocalyx* Kurz	Used to treat swelling, cancer, gastrointestinal disorders, and blood-related conditions	[34,35]
*Elaeagnus latifolia* L.	Used as a tonic and to treat muscle pain, cardiovascular disorders, skin diseases, gastrointestinal disorders, and fever	[33,34,35]
*Elaeocarpus hygrophilus* Kurz	Used to treat gynecological and urinary disorders and gastrointestinal disorders	[34]
*Feroniella lucida* (Scheff.) Swingle	Used to treat infections, fever, gynecological and urinary disorders, and blood-related conditions	[33,34]
*Finlaysonia pierrei* (Costantin) Venter	Used to treat fever	[34]
*Flacourtia indica* (Burm.f.) Merr.	Used to treat gastrointestinal disorders, infections, gynecological and urinary disorders, musculoskeletal disorders, and to promote lactation	[34,35,37]
*Garcinia cowa* Roxb. ex Choisy	Used to treat fever, musculoskeletal disorders, gastrointestinal disorders, and infections	[34]
*Hymenocardia punctata* Wall. ex Lindl.	Used to treat infections, poisoning, and musculoskeletal disorders	[34]
*Lepisanthes senegalensis* (Poir.) Leenh.	Used to treat fever and gastrointestinal disorders	[34]
*Madhuca thorelii* (Pierre ex Dubard) H.J.Lam	Used to treat gastrointestinal disorders and skin diseases	[34]
*Mangifera caloneura* Kurz	Used to treat gastrointestinal disorders	[34]
*Muntingia calabura* L.	Used as an antiseptic and anti-inflammatory agent and to treat gastric ulcers, swelling, headache, and cold	[40]
*Nephelium hypoleucum* Kurz	Used in postpartum care and to treat infections and blood-related conditions	[34,37]
*Parinari anamensis* Hance	Used as a tonic and to treat swelling, respiratory disorders, and skin diseases	[33,34,35]
*Passiflora foetida* L.	Used to treat diarrhea, hypertension, heart disease, respiratory disorders, and blood-related conditions	[33,34,35,36]
*Pithecellobium dulce* (Roxb.) Benth.	Used to treat lymphatic disorders, infections, skin diseases, and blood-related conditions	[34]
*Protium serratum* (Wall. ex Colebr.) Engl.	Used to treat fever	[34]
*Salacia chinensis* L.	Used to treat gastrointestinal disorders, diarrhea, constipation, kidney detoxification, musculoskeletal disorders, gynecological and urinary disorders, and infections	[33,34,35,37]
*Schleichera oleosa* (Lour.) Oken	Used as a laxative to relieve constipation	[36,37]
*Sindora siamensis* Teijsm. ex Miq.	Used to treat musculoskeletal disorders, pain, and gastrointestinal disorders	[33,34,35]
*Suregada multiflora* (A.Juss.) Baill.	Used to treat respiratory disorders, lymphatic disorders, gynecological and urinary disorders, cancer, and blood-related conditions	[32,33]
*Syzygium antisepticum* (Blume) Merr. & L.M.Perry	Used to treat pregnancy-related conditions, jaundice, poisoning, and skin diseases	[33,35]
*Syzygium cumini* (L.) Skeels	Used to treat gastrointestinal disorders, infections, and gynecological and urinary disorders	[34]
*Terminalia chebula* Retz.	Used to treat gastrointestinal disorders, constipation, respiratory conditions, cardiovascular disorders, gynecological and urinary disorders, skin diseases, and infections	[33,34,41]
*Uvaria ferruginea* var. *cherrevensis* (Pierre ex Finet & Gagnep.) Meade & J.Parn.	Used to treat hemorrhoids, stomach pain, fever, and gynecological and urinary disorders	[34,37]
*Uvaria rufa* Blume	Used to treat gynecological and urinary disorders, fever, and poisoning	[34]
*Willughbeia edulis* Roxb.	Used to treat circulatory disorders, infections, skin diseases, gastrointestinal disorders, and blood-related conditions	[34,37]
*Xylia xylocarpa* (Roxb.) W.Theob.	Used to treat musculoskeletal disorders, infections, gastrointestinal disorders, pain, cancer, and gynecological and urinary disorders	[33,34,37]
*Ziziphus mauritiana* Lam.	Used to treat gastrointestinal disorders, infections, and fever	[34]
*Ziziphus oenopolia* (L.) Mill.	Used to treat diabetes, hemorrhoids, muscle pain, abscesses, urinary disorders, constipation, and blood-related conditions	[33,34,35,37,41]

## Data Availability

The data presented in this study are available on request from the first authors or corresponding authors.

## Supplementary Materials

The following supporting information can be downloaded at: https://www.mdpi.com/article/10.3390/biology15090711/s1, Table S1: Use reports and Cultural Importance Index (CI) values of wild edible fruit species recorded in Kut Chum District, Yasothon Province, Thailand.

## References

[1] Kumar A., Kumar S., Komal, Ramchiary N., Singh P. (2021). Role of Traditional Ethnobotanical Knowledge and Indigenous Communities in Achieving Sustainable Development Goals. Sustainability.

[2] Lulesa F., Alemu S., Kassa Z., Awoke A. (2025). Ethnobotanical Investigation of Medicinal Plants Utilized by Indigenous Communities in the Fofa and Toaba Sub-Districts of the Yem Zone, Central Ethiopian Region. J. Ethnobiol. Ethnomedicine.

[3] Yao R., He C., Xiao P. (2022). ‘Food and Medicine Continuum’ in the East and West: Old Tradition and Current Regulation. Chin. Herb. Med..

[4] Alemayehu G., Awoke A., Kassa Z. (2025). Wild Edible Plant Species and Their Role in Nutrition and Health in Korahe Zone, Eastern Ethiopia. Trop. Med. Health.

[5] González-Zamorano L., Cámara R.M., Morales P., Cámara M. (2025). Harnessing Edible Wild Fruits: Sustainability and Health Aspects. Nutrients.

[6] Najmi A., Javed S.A., Al Bratty M., Alhazmi H.A. (2022). Modern Approaches in the Discovery and Development of Plant-Based Natural Products and Their Analogues as Potential Therapeutic Agents. Molecules.

[7] Sun W., Shahrajabian M.H. (2023). Therapeutic Potential of Phenolic Compounds in Medicinal Plants—Natural Health Products for Human Health. Molecules.

[8] Nunes C.d.R., Barreto Arantes M., Menezes de Faria Pereira S., Leandro da Cruz L., de Souza Passos M., Pereira de Moraes L., Vieira I.J.C., Barros de Oliveira D. (2020). Plants as Sources of Anti-Inflammatory Agents. Molecules.

[9] Agu P.C., Nduneseokwu N.C., Nwiziogo F.C., Okafor M.U., Alum E.U., Egwu C.O., Anidu I.C., Ezinwa C.A., Ibiam A.U., Aja P.M. (2025). Historical and Ethnopharmacological Perspectives on African Medicinal Plants: From Traditional Remedies to Computational Drug Discovery. Sci. Afr..

[10] Hassan M., Mir T.A., Jan M., Amjad M.S., Aziz M.A., Pieroni A., Vitasović-Kosić I., Bussmann R.W. (2024). Foraging for the future: Traditional culinary uses of wild plants in the Western Himalayas–Kashmir Valley (India). J. Ethnobiol. Ethnomed..

[11] Mothupi F.M., Shackleton C.M. (2025). Traditional Knowledge and Consumption of Wild Edible Plants in Rural Households, Limpopo Province, South Africa. J. Ethnobiol. Ethnomed..

[12] Pawera L., Khomsan A., Zuhud E.A.M., Hunter D., Ickowitz A., Polesny Z. (2020). Wild Food Plants and Trends in Their Use: From Knowledge and Perceptions to Drivers of Change in West Sumatra, Indonesia. Foods.

[13] Alrhmoun M., Guiggi V., Gillani S.W., Manzoor M., Sulaiman N., Pieroni A. (2025). Diachronic Changes in Local Food Heritage: The Ethnobiology of Wild Foods in Central Tuscany. J. Ethnobiol. Ethnomedicine.

[14] Saensouk P., Saensouk S., Appamaraka S., Koompoot K., Sengthong A., Phengmala K., Jitpromma T. (2026). Utilization of Wild Edible Plants by the Tai Yoy Ethnic Group in Akat Amnuai District, Sakon Nakhon Province, Thailand. Biology.

[15] Saensouk P., Saensouk S., Sonthongphithak P., Junsongduang A., Koompoot K., Huang B., Shen W., Jitpromma T. (2026). Ethnobotany of Local Vegetables and Spices in Sakon Nakhon Province, Thailand. Diversity.

[16] Ul Abidin S.Z., Khan R., Ahmad M., Cuerrier A., Zafar M., Ullah A., Khan J., Saeed A., Al-Qahtani W.H., Kazi M. (2024). Wild Edible Fruits as a Source of Food and Medicine: A Study among Tribal Communities of Southern Khyber Pakhtunkhwa. Plants.

[17] Parnwell M.J.G. (2007). Neolocalism and Renascent Social Capital in Northeast Thailand. Environ. Plan. D Soc. Space.

[18] QGIS Development Team (2023). QGIS Geographic Information System. Open Source Geospatial Foundation Project. https://qgis.org.

[19] Piyavaranon P., Panpakdee C. (2025). Exploring Factors Influencing the Adoption of the Khok Nong Na Model for Sustainable Agriculture in Northeast Thailand. Discov. Sustain..

[20] Aumtong S., Somyo C., Kanchai K., Chuephudee T., Chotamonsak C. (2025). Relationships Between Carbon Fractions and Soil Nutrients in Organic Cassava Cultivation in the Sandy Soil of Northeastern Thailand. Agronomy.

[21] Kheoruenromne I., Suddhiprakarn A., Kanghae P. (1998). Properties, environment and fertility capability of sandy soils in Northeast Plateau, Thailand. Agric. Nat. Resour..

[22] Hereward J.P., Cai X., Matias A.M.A., Walter G.H., Xu C., Wang Y. (2020). Migration dynamics of an important rice pest: The brown planthopper (*Nilaparvata lugens*) across Asia—Insights from population genomics. Evol. Appl..

[23] Arunrat N., Kongsurakan P., Sereenonchai S., Hatano R. (2020). Soil Organic Carbon in Sandy Paddy Fields of Northeast Thailand: A Review. Agronomy.

[24] Muthu Narayanan M., Ahmad N., Shivanand P., Metali F. (2022). The Role of Endophytes in Combating Fungal- and Bacterial-Induced Stress in Plants. Molecules.

[25] Plants of the World Online (POWO). Facilitated by the Royal Botanic Gardens, Kew. Published on the Internet. https://powo.science.kew.org/.

[26] Tajik O., Golzar J., Noor S. (2025). Purposive Sampling. Int. J. Educ. Learn. Stud..

[27] International Society of Ethnobiology (ISE) (2006). International Society of Ethnobiology Code of Ethics (with 2008 Additions). https://www.ethnobiology.net/what-we-do/core-programs/ise-ethics-program/code-of-ethics/.

[28] Secretariat of the Convention on Biological Diversity (2011). Nagoya Protocol on Access to Genetic Resources and the Fair and Equitable Sharing of Benefits Arising from Their Utilization to the Convention on Biological Diversity.

[29] Saensouk P., Saensouk S., Boonma T., Zhang Y., Lv L., Jitpromma T. (2025). Ethnobotanical Heritage of Edible Plants Species in Mueang District, Yasothon Province, Northeastern Thailand. Biology.

[30] Tardío J., Pardo-de-Santayana M. (2008). Cultural Importance Indices: A Comparative Analysis Based on the Useful Wild Plants of Southern Cantabria (Northern Spain). Econ. Bot..

[31] Friedman J., Yaniv Z., Dafni A., Palewitch D. (1986). A preliminary classification of the healing potential of medicinal plants, based on a rational analysis of an ethnopharmacological field survey among Bedouins in the Negev Desert, Israel. J. Ethnopharmacol..

[32] Heinrich M., Ankli A., Frei B., Weimann C., Sticher O. (1998). Medicinal plants in Mexico: Healers’ consensus and cultural importance. Soc. Sci. Med..

[33] Junsongduang A., Saensouk S., Balslev H. (2025). Amnat Charoen healers in Thailand and their medicinal plants. Plants.

[34] Saensouk P., Saensouk S., Boonma T., Junsongduang A., Naing M.K., Jitpromma T. (2025). Ethnomedicinal properties of wild edible fruit plants and their horticultural potential among indigenous Isan communities in Roi Et Province, Northeastern Thailand. Horticulturae.

[35] Junsongduang A., Kasemwan W., Lumjoomjung S., Sabprachai W., Tanming W., Balslev H. (2020). Ethnomedicinal knowledge of traditional healers in Roi Et, Thailand. Plants.

[36] Niamngon P., Saensouk S., Saensouk P., Junsongduang A. (2023). Ethnobotanical knowledge of Isaan Laos tribe in Khong Chai District, Kalasin Province, Thailand with particular focus on medicinal uses. Biodiversitas.

[37] Saisor N., Prathepha P., Saensouk S. (2021). Ethnobotanical study and utilization of plants in Khok Nhong Phok forest, Kosum Phisai District, Northeastern Thailand. Biodiversitas.

[38] Lumlerdkij N. (2018). Thai Traditional Medicine as a Source for Cancer Prevention: from Local Concepts to the Discovery of Potential Chemopreventive Extracts. Ph.D. Thesis.

[39] Mondal M., Konar A., Halder S., Roy A., Dalal D.D., Ghosh P. (2024). An insight into the morphological, ethnomedicinal, phytochemical and pharmaceutical properties of *Buchanania lanzan*. J. Med. Plants Stud..

[40] Mahmood N.D., Nasir N.L.M., Rofiee M.S., Tohid S.F.M., Ching S.M., Teh L.K., Zakaria Z.A. (2014). *Muntingia calabura*: A review of its traditional uses, chemical properties, and pharmacological observations. Pharm. Biol..

[41] Saensouk P., Saensouk S., Huang B., Shen W., Chanthavongsa K., Junsongduang A., Sonthongphithak P., Jitpromma T. (2026). Ethnomedicinal plants and traditional healing practices of indigenous communities in Dan Sub-district, Kap Choeng District, Surin Province, Thailand. J. Ethnobiol. Ethnomed..

[42] Suwardi A.B., Syamsuardi, Mukhtar E., Nurainas (2023). The diversity and traditional knowledge of wild edible fruits in Bengkulu, Indonesia. Ethnobot. Res. Appl..

[43] Suwardi A.B., Syamsuardi, Mukhtar E., Nurainas (2023). Ethnobotany and conservation of wild edible fruits in Sumatra: A case study in Pesisir Selatan, West Sumatra, Indonesia. Philipp. J. Sci..

[44] Kidane B., van der Maesen L., van Andel T., Asfaw Z., Sosef M. (2014). Ethnobotany of wild and semi-wild edible fruit species used by Maale and Ari ethnic communities in southern Ethiopia. Ethnobot. Res. Appl..

[45] Mgalula M.E. (2024). An ethnobotanical study of wild edible fruits in miombo woodlands of Tabora region in western Tanzania. J. Ethnobiol. Ethnomed..

[46] Bhavana N.S., Shrishail, Masarbo R.S., Bindu D.N. (2024). Ethnobotanical survey of wild edible fruits from two selected regions of Karnataka, India. J. Med. Plants Stud..

[47] Vinceti B., Termote C., Ickowitz A., Powell B., Kehlenbeck K., Hunter D. (2013). The Contribution of Forests and Trees to Sustainable Diets. Sustainability.

[48] Bhatt H., Pant Jugran H., Pandey R. (2024). Cultural ecosystem services nexus with socio-cultural attributes and traditional ecological knowledge for managing community forests of Indian western Himalaya. Ecol. Indic..

[49] Esperon-Rodriguez M. (2025). Trees, society, and the path toward resilient ecosystems. Plants People Planet.

[50] Reed J., van Vianen J., Foli S., Clendenning J., Yang K., MacDonald M., Petrokofsky G., Padoch C., Sunderland T. (2017). Trees for life: The ecosystem service contribution of trees to food production and livelihoods in the tropics. For. Policy Econ..

[51] Jessen T.D., Ban N.C., Claxton N.X., Darimont C.T. (2022). Contributions of Indigenous knowledge to ecological and evolutionary understanding. Front Ecol. Environ..

[52] Ijatuyi E.J., Lamm A., Yessoufou K., Suinyuy T., Patrick H.O. (2025). Integration of indigenous knowledge with scientific knowledge: A systematic review. Environ. Sci. Policy.

[53] Negi V.S., Pathak R., Thakur S., Joshi R.K., Bhatt I.D., Rawal R.S. (2023). Scoping the need of mainstreaming indigenous knowledge for sustainable use of bioresources in the Indian Himalayan region. Environ. Manag..

[54] Zaca F.N., Chipfupa U., Ojo T.O., Managa L.R., Mabhaudhi T., Slotow R., Ngidi M.S.C. (2025). The role of fruit trees in reducing food insecurity and improving nutrition security of rural households: A case study of the KwaZulu-Natal Province, South Africa. J. Agric. Food Res..

[55] Rumicha T.D., Belew S., Hasen G., Teka T.A., Forsido S.F. (2025). Food, feed, and phytochemical uses of wild edible plants: A systematic review. Food Sci. Nutr..

[56] Kumar B.M., Bhavya G., De Britto S., Jogaiah S. (2025). Wild edible plants for food security, dietary diversity, and nutraceuticals: A global overview of emerging research. Front. Sustain. Food Syst..

[57] Harmayani E., Anal A.K., Wichienchot S., Bhat R., Gardjito M., Santoso U., Siripongvutikorn S., Puripaatanavong J., Payyappallimana U. (2019). Healthy food traditions of Asia: Exploratory case studies from Indonesia, Thailand, Malaysia, and Nepal. J. Ethn. Food.

[58] Peduruhewa P.S., Jayathunge K.G.L.R., Liyanage R. (2021). Potential of underutilized wild edible plants as food for the future: A review. J. Food Secur..

[59] Saensouk P., Saensouk S., Chanthavongsa K., Sonthongphithak P., Jitpromma T. (2025). Ornamental Plant Diversity and Traditional Uses in Home Gardens of Kham Toei Sub-District, Thai Charoen District, Yasothon Province, Northeastern Thailand. Diversity.

[60] Luo B., Tong Y., Liu Y., Zhang Y., Qin Y., Hu R. (2024). Ethnobotanical insights into the traditional food plants of the Baiku Yao community: A study of cultural significance, utilization, and conservation. J. Ethnobiol. Ethnomed..

[61] Zhang S., He C., Su L., Wang H., Lin J., Li Y. (2025). An ethnobotanical study on wild edible plants in Taishan County, Guangdong, China. Front. Sustain. Food Syst..

[62] Gebre A., Gitima G., Berhanu Y. (2025). Ethnobotanical study of wild edible plants in Goba District Southwest Ethiopia. Sci. Rep..

[63] Tadesse T., Teka A. (2023). Ethnobotanical study on medicinal plants used by the local communities of Ameya District, Oromia Regional State, Ethiopia. Evid.-Based Complement. Altern. Med..

[64] Wani Z.A., Shreekar P., Bikarma S. (2021). Descriptive study of plant resources in the context of the ethnomedicinal relevance of indigenous flora; a case study from Rajouri-Poonch region of Himalaya. Ethnobot. Res. Appl..

[65] Alrhmoun M., Sulaiman N., Pieroni A. (2025). Shifting Herbal Knowledge: The Ecological and Cultural Dynamics Behind Plant Use Changes in the Southern Occitan Alps. Plants.

[66] Alrhmoun M., Sulaiman N., Pieroni A. (2024). What Drives Herbal Traditions? The Influence of Ecology and Cultural Exchanges on Wild Plant Teas in the Balkan Mountains. Land.

[67] Ramdé S.J.C., Ganamé M., Bayen P., Lykke A.M., Thiombiano A. (2025). Impact of urbanization on uses and conservation of indigenous leafy vegetable species in Burkina Faso, West Africa. J. Agric. Food Res..

[68] Malapane O.L., Chanza N., Musakwa W. (2024). Transmission of indigenous knowledge systems under changing landscapes within the Vhavenda community, South Africa. Environ. Sci. Policy.

[69] Bański J. (2025). The role of knowledge transferred between rural inhabitants and newcomers in the development of rural areas. Rural Reg. Dev..

[70] Tang R., Gavin M.C. (2016). A classification of threats to traditional ecological knowledge and conservation responses. Conserv. Soc..

[71] Brindis-Badillo D.A., Arroyo-Rodríguez V., Mendoza E., Wies G., Martínez-Ramos M. (2022). Conserving dominant trees in human-modified landscapes at the Lacandon tropical rainforest. Biol. Conserv..

[72] Rocha-Santos L., Mayfield M.M., Lopes A.V., Pessoa M.S., Talora D.C., Faria D., Cazetta E. (2020). The loss of functional diversity: A detrimental influence of landscape-scale deforestation on tree reproductive traits. J. Ecol..

[73] Freitas C.T., Lopes P.F.M., Campos-Silva J.V., Noble M.M., Dyball R., Peres C.A. (2020). Co-management of culturally important species: A tool to promote biodiversity conservation and human well-being. People Nat..

[74] Lukawiecki J., Moola F., Roth R. (2024). Cultural keystone species and their role in biocultural conservation. Conserv. Sci. Pract..

[75] Min Q., Yang X., Ding L. (2022). The concept, connotation and significance of cultural keystone species in agricultural heritage systems. J. Resour. Ecol..

[76] Petelka J., Bonari G., Säumel I., Plagg B., Zerbe S. (2022). Conservation with local people: Medicinal plants as cultural keystone species in the Southern Alps. Ecol. Soc..

[77] Wang L., Wei F., Tagesson T., Fang Z., Svenning J.-C. (2025). Transforming forest management through rewilding: Enhancing biodiversity, resilience, and biosphere sustainability under global change. One Earth.

[78] Sangha K.K., Leyton-Flor S.A., Conner N., Bhardwaj A., Bhardwaj A.K. (2025). Transforming conservation by understanding the role of Indigenous peoples and local communities and their economies. Glob. Ecol. Conserv..

[79] Hu X., Bai L., Wang M., Chen Q., Xu C., Long C. (2026). Traditional ecological knowledge of wild edible plants in the Dai communities of Lujiangba area, western Yunnan, China. J. Ethnobiol. Ethnomed..

[80] Bajgai R.C., Bajgai Y., Johnson S.B. (2023). The presence of wild edible plants and determinants influencing their harvest, consumption, and conservation in southeastern Bhutan. PLoS ONE.

