# Supplementary material for: Ethnobotany and Medicinal Potential of Wild Edible Fruit Species in Kut Chum District, Yasothon Province, Thailand
Source: Biology (Basel). 2026 Apr 30;15(9):711. doi: 10.3390/biology15090711 (PMC13163093; doi:10.3390/biology15090711)
Supplement: Supplementary file 1 [file biology-15-00711-s001.zip › biology-4285591-supplementary.pdf]

biology

MDPI

Supplementary

Ethnobotany and Medicinal Potential of Wild Edible Fruit Species in Kut Chum District, Yasothon Province, Thailand

Table S1. Use reports and Cultural Importance Index (CI) values of wild edible fruit species recorded in Kut Chum District, Yasothon Province, Thailand.

Scientific name	Use-reports				$CI_{categories}$				CI
	BV	FT	MD	FC	BV	FT	MD	FC	
Acronychia pedunculata (L.) Miq.	-	9	12	-	-	0.150	0.200	-	0.350
Alyxia schlechteri H.Lév.	-	9	-	-	-	0.150	-	-	0.150
Ampelocissus martini Planch.	-	31	15	-	-	0.517	0.250	-	0.767
Antidesma ghaesembilla Gaertn.	6	32	-	-	0.100	0.533	-	-	0.633
Artabotrys spinosus Craib	-	13	-	-	-	0.217	-	-	0.217
Artocarpus lacucha Buch.-Ham.	-	37	-	-	-	0.617	-	-	0.617
Azima sarmentosa (Blume) Benth. & Hook.f.	-	12	-	-	-	0.200	-	-	0.200
Buchanania lanzan Spreng.	-	23	-	-	-	0.383	-	-	0.383
Buchanania siamensis Miq.	-	-	12	-	-	-	0.200	-	0.200
Cajanus cajan (L.) Huth	-	22	-	-	-	0.367	-	-	0.367
Calamus caesius Blume	-	21	-	-	-	0.350	-	-	0.350
Cananga brandisiana (Pierre) Saff.	-	-	11	-	-	-	0.183	-	0.183
Canarium subulatum Guillaumin	-	27	-	-	-	0.450	-	-	0.450
Canthium berberidifolium E.T.Geddes	-	6	10	-	-	0.100	0.167	-	0.267
Castanopsis piriformis Hickel & A.Camus	-	23	-	-	-	0.383	-	-	0.383
Dasymaschalon lomentaceum Finet & Gagnep.	-	15	-	-	-	0.250	-	-	0.250
Dialium cochinchinense Pierre	-	29	-	-	-	0.483	-	-	0.483
Dillenia ovata Wall. ex Hook.f. & Thomson	-	27	-	-	-	0.450	-	-	0.450
Diospyros decandra Lour.	-	36	-	-	-	0.600	-	-	0.600
Diospyros filipendula Pierre ex Lecomte	-	-	20	-	-	-	0.333	-	0.333
Diospyros rhodocalyx Kurz	-	12	-	-	-	0.200	-	-	0.200
Elaeagnus latifolia L.	-	11	-	-	-	0.183	-	-	0.183
Elaeocarpus hygrophilus Kurz	-	32	-	-	-	0.533	-	-	0.533
Feroniella lucida (Scheff.) Swingle	-	8	-	-	-	0.133	-	-	0.133
Ficus hispida L.f.	-	21	9	-	-	0.350	0.150	-	0.500
Ficus racemosa L.	-	20	11	-	-	0.333	0.183	-	0.517
Finlaysonia pierrei (Costantin) Venter	-	27	-	-	-	0.450	-	-	0.450
Flacourtia indica (Burm.f.) Merr.	-	37	-	-	-	0.617	-	-	0.617
Garcinia cowa Roxb. ex Choisy	-	16	-	-	-	0.267	-	-	0.267
Goniothalamus laoticus (Finet & Gagnep.) Bân	-	-	10	-	-	-	0.167	-	0.167

Grewia hirsuta Vahl	-	-	13	-	-	-	0.217	-	0.217
Huberantha cerasoides (Roxb.) Chaowasku	-	-	11	-	-	-	0.183	-	0.183
Hydnocarpus castaneus Hook.f. & Thomson	-	5	8	-	-	0.083	0.133	-	0.217
Hymenocardia punctata Wall. ex Lindl.	-	18	-	-	-	0.300	-	-	0.300
Irvingia malayana Oliv. ex A.W.Benn.	-	37	15	-	-	0.617	0.250	-	0.867
Lepisanthes rubiginosa (Roxb.) Leenh.	-	26	8	-	-	0.433	0.133	-	0.567
Lepisanthes senegalensis (Poir.) Leenh.	-	19	-	-	-	0.317	-	-	0.317
Limonia acidissima L.	-	15	6	-	-	0.250	0.100	-	0.350
Madhuca thorelii (Pierre ex Dubard) H.J.Lam	-	22	-	-	-	0.367	-	-	0.367
Mangifera caloneura Kurz	-	32	-	-	-	0.533	-	-	0.533
Melastoma malabathricum L.	-	-	13	-	-	-	0.217	-	0.217
Memecylon edule Roxb.	-	-	10	-	-	-	0.167	-	0.167
Microcos tomentosa Sm.	-	24	12	-	-	0.400	0.200	-	0.600
Muntingia calabura L.	-	32	-	-	-	0.533	-	-	0.533
Nephelium hypoleucum Kurz	-	37	-	-	-	0.617	-	-	0.617
Parinari anamensis Hance	-	17	-	-	-	0.283	-	-	0.283
Passiflora foetida L.	-	16	-	-	-	0.267	-	-	0.267
Phyllanthus emblica L.	-	35	15	-	-	0.583	0.250	-	0.833
Pithecellobium dulce (Roxb.) Benth.	-	34	-	-	-	0.567	-	-	0.567
Polyalthia debilis (Pierre) Finet & Gagnep.	-	11	8	-	-	0.183	0.133	-	0.317
Polyalthia evecta (Pierre) Finet & Gagnep.	-	21	9	-	-	0.350	0.150	-	0.500
Protium serratum (Wall. ex Colebr.) Engl.	-	18	-	-	-	0.300	-	-	0.300
Salacia chinensis L.	-	19	-	-	-	0.317	-	-	0.317
Schleichera oleosa (Lour.) Oken	-	39	-	-	-	0.650	-	-	0.650
Sindora siamensis Teijsm. ex Miq.	-	20	-	-	-	0.333	-	-	0.333
Streblus asper Lour.	-	22	14	-	-	0.367	0.233	-	0.600
Suregada multiflora (A.Juss.) Baill.	-	13	-	-	-	0.217	-	-	0.217
Syzygium antisepticum (Blume) Merr. & L.M.Perry	-	31	-	-	-	0.517	-	-	0.517
Syzygium cumini (L.) Skeels	5	35	-	-	0.083	0.583	-	-	0.667
Terminalia chebula Retz.	-	26	-	-	-	0.433	-	-	0.433
Urceola polymorpha (Pierre ex Spire) D.J.Middleton & Livsh.	-	-	10	19	-	-	0.167	0.317	0.483
Uvaria dulcis Dunal	-	13	7	-	-	0.217	0.117	-	0.333
Uvaria ferruginea Buch.-Ham. ex Hook.f. & Thomson	-	12	11	-	-	0.200	0.183	-	0.383
Uvaria ferruginea var. *cherrevensis* (Pierre ex Finet & Gagnep.) Meade & J.Parn.	-	16	-	-	-	0.267	-	-	0.267
Uvaria rufa Blume	-	38	-	-	-	0.633	-	-	0.633
Uvaria siamensis (Scheff.) L.L.Zhou, Y.C.F.Su & R.M.K.Saunders	-	-	19	-	-	-	0.317	-	0.317
Willughbeia edulis Roxb.	-	35	-	-	-	0.583	-	-	0.583

Xylia xylocarpa (Roxb.) W.Theob.	-	21	-	-	-	0.350	-	-	0.350
Xylopia vielana Pierre	-		18	-	-	-	0.300	-	0.300
Ziziphus mauritiana Lam.	-	33	-	-	-	0.550	-	-	0.550
Ziziphus oenopolia (L.) Mill.	2	36	-	-	0.033	0.600	-	-	0.633
Total	13	1384	317	19	0.217	23.067	5.283	0.317	

Abbreviation. Utilization: BV (beverages), FT (fruit), MD (medicine), SP (food ingredient or culinary additive).
